# Using player types to understand cooperative behaviour under economic and sociocultural heterogeneity in common-pool resources: Evidence from lab experiments and agent-based models

**DOI:** 10.1371/journal.pone.0268616

**Published:** 2022-05-25

**Authors:** Fijnanda van Klingeren

**Affiliations:** Rotterdam School of Management, Erasmus University Rotterdam, Rotterdam, The Netherlands; Groupe ESC Dijon Bourgogne, FRANCE

## Abstract

Rising migration numbers and the resulting increase in economic and sociocultural heterogeneity in societies all over the world are theorised to put pressure on the sustainable use of common-pool resources [CPRs]. Increased heterogeneity is argued to decrease trust and diversify interests between resource users, leading to overuse and decline of natural and man-made CPRs. The aim of this paper is to understand cooperative behaviour under economic and sociocultural heterogeneity in CPRs, through the analyses of experimental data including 344 subjects from the United Kingdom and the Netherlands, and 144 subjects from India. Multilevel regression, ordinal logistic regression, linear conditional-contribution profiles [LCPs] and agent-based models [ABMs] are used to analyse and replicate experimental outcomes on the micro- and macro-level. Results show that the combination of economic and sociocultural heterogeneity affects cooperation negatively when the decision-situation is perceived as unfair, but that neither economic nor sociocultural heterogeneity on themselves affect cooperation negatively. Economic heterogeneity is even found to affect cooperation positively relative to homogeneity. Player type classification based on LCP scores shows that experimental outcomes can be interpreted with player types, and ABM simulations validate the experimental results by replicating the main outcomes.

## Introduction

Over the past 50 years migration worldwide has been increasing steadily with almost 281 million people living in another country than their birth country in 2020—that’s 128 million people more than 30 years before [1]. Societies are becoming more diverse on sociocultural and economic dimensions, which may pose new challenges for cooperation dilemma contexts such as common-pool resources [CPRs]: man-made or natural resources used by many but owned by nobody in particular, such as common pastures, fishing grounds and irrigation systems [2]. The depletion and overuse of resources, amongst which many CPRs, is a growing problem [3]. The increasing heterogeneity amongst resource users—bringing with it a potential diversification of interests, more costly coordination and communication and reduced trust between resource users—may put even more stress on sustainable use of CPRs. However, the effect of sociocultural and economic heterogeneity on cooperation in CPRs is still understudied. The aim of this paper is to understand cooperative behaviour under economic and sociocultural heterogeneity in CPRs.

Economic heterogeneity, defined in this paper as heterogeneity in wealth or income, is generally found to decrease cooperation through diversification of interests of individuals [4–7]. On the other hand, there is research suggesting a positive effect of economic heterogeneity on cooperation through the “Olson-effect” [8] suggesting that under specific circumstances cooperation can be established in economically heterogeneous groups, in a situation in which the rich bear the cost of cooperation for the poor.

Sociocultural heterogeneity, defined in this paper as heterogeneity in language, ethnicity, religion or other cultural expressions [9–11], is generally found to reduce cooperation as individuals are more likely to cooperate with others that are similar to themselves [12–21].

Another variable that is deemed important in this context is trust, as literature and empirical results suggests that individuals trust others that are like them, for instance with respect to ethnicity, culture or religion [22–27]. Trust in its turn has been widely recognised to stimulate cooperation [28–32].

To find empirical evidence on the positive or negative relation between economic and sociocultural heterogeneity, trust and cooperation, this paper explores the role that methodological tools like player type classification and simple agent-based models [ABMs] can play in the analysis of behavioural experimental data. The data analysed and compared in this paper come from two CPR experiments; laboratory experiments mimicking a CPR cooperation dilemma in which subjects have to make decisions on subtraction from a resource that is used by multiple players. The first experiment—hereafter called the ‘UKNL study’—is presented in Van Klingeren [31] and was conducted in the United Kingdom and the Netherlands with 344 subjects. The second experiment—hereafter called the ‘IND study’—is presented in this paper for the first time and was conducted in India with 144 subjects. The latter experiment is a replication study and robustness check for the former experiment presented in Van Klingeren [31].

The importance of studying CPRs with specific CPR experiments—as opposed to other tools to analyse cooperative group behaviour such as Public Good [PG] games—lies in the unique form of the cooperation dilemma in CPRs. Whereas PGs and CPRs have in common that it is hard to exclude potential users, an additional key characteristic of CPRs is their high subtractability [18]; the resource may run out and cease to exist. This in combination with their non-excludability makes CPRs vulnerable to the ‘Tragedy of the Commons’ as famously described by Hardin [33]: a situation in which the CPR is confined into a system that provides each resource-user the motivation to use the limited resource unlimitedly, which will lead to its unavoidable deterioration and depletion. Although many studies point out that CPRs do not have to end in tragedy, for instance when they use institutions for collective action to guide user behaviour [2, 34–36], this type of dilemma is unique to CPRs and relevant to classic commons such as fisheries, forests and pastures as well as new forms of commons such as citizen collectives for food, green energy and urban agriculture projects [34, 35, 37]. Both classic and contemporary forms of CPRs will face increased heterogeneity of users, clients and members, and often struggle with the same cooperation dilemmas.

This paper continues with a discussion of the experimental design, data and methods. Then the results are presented and discussed and suggestions for future research are proposed. The paper ends with stating some final conclusions. The findings will advance theoretical insights on cooperation under heterogeneity in CPRs as well as provide proof and recommendations on treatment operationalisation in behavioural experiments and the use of innovative computational tools for the analysis of experimental data.

## Materials & methods

The methodological strategy of this paper comprises statistical analyses of experimental data, player type classification through the construction of linear conditional-contribution profile [LCP] scores as described and applied by Kurzban & Houser [38] and replicating experimental outcomes with ABMs following Janssen & Baggio [39]. All three methods of analysis add to the understanding of cooperative behaviour under heterogeneity in their own way. Firstly, the statistical analysis of the experimental data establishes significant differences between behaviour in different heterogeneity treatments, both on the macro- and micro-level. Secondly, player type classification is useful for identifying and visualising the emergence of different behavioural types in different treatments, and to express experimental outcomes as a result of player type distribution. It will provide a more granular insight on behavioural differences on the player-level. Thirdly, ABMs based on simple theoretical agents are used in accordance with the earlier established player type distribution from the data to replicate and explain experimental outcomes on the micro- and macro-level.

In the following chapter, the experimental design of the two studies is discussed, after which the three methodologies that are used in this paper are presented and explained in detail.

### The experiment

The first part of both experiments comprised a (1) one-shot Investment Game [IG] [40] to measure general trust; (2) a basic, practise version of the CPR game called ‘the Fishing Game’ without any treatments for three periods; and (3) a group division stage. In the second part of the experiments, the subjects started the Fishing Game with heterogeneity treatments. Details on the course of the experimental sessions can be found in S1 Text. A power analysis to determine the sample size for the IND study can be found in S2 Text. This research, including the pre-tests, has obtained ethics clearance from the Research Ethics Committee of Department of Sociology (DREC) at the University of Oxford (Ref. SOC_R2_001_C1A_18_30), the CESS ethics committee (Ref. LE_0044) covering both the UKNL and IND study as they are both part of the same experimental centre and have the same ethical standards, rules and procedures. The sessions in the Netherlands were in addition covered by the ethical approval (Ref. FETC17–028, Buskens) of the ethical committee of the Faculty of the Social and Behavioural Sciences at Utrecht University. All participants were of age 18 or older, no deception was used in the experimental setup and written consent was obtained from all subjects in the study, including the pre-test, before the start of each experimental session. The data was anonymised before the analyses. A form detailing the current paper in light of inclusiveness in global research can be found in S3 Text.

#### The Fishing game: Cooperation and heterogeneity

A detailed explanation of each stage of the Fishing Game including utility and regrowth functions can be found in S4 Text and in Van Klingeren [31]. In short, the game is played as follows. The resource in both experiments is a fishing ground. In the game there are four resource users or ‘appropriators’ that use the CPR. They play the game with each other for the entire session. During the game, players can invest all or part of their endowment *E* in the extraction or ‘appropriation’ of fish from the resource (i.e. they go fishing) for profit every period *t*. The resource regrows after each period to a certain extent, but consistent overappropriation will reduce the resource size over time. Profit per invested unit in appropriation *a* depends on the size of the resource relative to its original size. Cooperation in the experiment is operationalised as the appropriation effort (lower is more cooperative, as can be learned from the experiment description in S4 Text) and resource size (higher is more cooperative) and heterogeneity is operationalised with treatments of economic and/or sociocultural heterogeneity. The game lasts 40 periods in the UKNL study, and 30 periods in the IND study. To be able to compare the data from both experiments, only the first 30 periods of the UKNL study are taken into account in the comparative analyses of this paper. Subjects do not know how many periods they will play the game for in either experiment, which means that no endgame effects are expected [41] and the first 30 periods are thus comparable between the two experiments.

#### Trust

In the UKNL study trust is measured at three points in the experiment: with a general IG, an IG after the division in groups—once with an ingroup and once with an outgroup member—and at the post-experimental survey. Due to time constraints in the IND study, the second trust measurement was omitted. Thus, we look at two points of measurement in the experimental sessions: (1) by the one-shot IG with a random other subject at the very beginning of the experimental session before the main game, and (2) by asking questions on the extent of mutual trust felt during the main game in a post-experimental survey. The one-shot IG will be used to measure general trust; to get an idea on how trusting the participants are when they enter the experiment. See S5 Text for an extensive explanation of the IG as played in the experiment. The post-experimental survey questions are analysed to see if there are significant differences in post-experimental reporting on trust between treatments.

### Experimental treatments

#### Sociocultural heterogeneity: Paintings and cities

In the UKNL study, a Minimal Group Experiment [MGE] was used to create artificial identities which are based on an intended trivial criterion [42–47]. Following the approach of amongst others Tajfel, Billig, Bundy and Flament [43], Aksoy [44, 48] and Kahn [46], the subjects were shown five paintings by two artists, Paul Klee and Wassily Kandinsky, after which they were asked to express their preference of either picture, resulting in a score of 0 to 5 for Klee-preference. Based on the median preference of the particular experimental session, subjects are assigned to the Klee or Kandinsky group [49]. To strengthen the group bonds, subjects play a quiz in which group performance pays off and an other-other Dictator Game. See S1 Table for the decision table of the other-other DG and Van Klingeren [31] for a more extensive explanation of this stage.

In the IND study, a real form of identity was used to operationalise sociocultural heterogeneity, namely whether subjects came originally from Mumbai or Bangalore. To make sure participants of the IND study were really from either city, subjects’ home towns were checked during the sign up process, and they were asked to provide some type of proof of their affiliation with either city during the registration process. Of course, one can never be 100% certain that someone who claims to identify strongly as a Mumbaikar based on their time spent there or coming out of the general area, but was not born there, really has the affiliation they say they have. However, Mumbaikars and Bangaloreans are randomly spread over treatments, so this should not influence treatment effects. A few students were not admitted to participate due to lack of or unconvincing “evidence” of their affiliation with either city.

From the answers to the open question of why subjects feel a connection to their city, it is clear that subjects felt passionate about their city, with some of the answers being:“*I am extremely influenced by its culture, people and ways of life*.”,“*It plays a big role in what I am today*”,“*[…] I’ve been socialized into the culture of the city*” and “*It is my home and when people meet me they know I’m from this city*”. In addition, half of the subjects were asked a question in the post-experimental survey on how strongly they identify with their city of origin on a 7-point Likert-scale. The results show that subjects from both Bangalore and Mumbai reported similar levels of identification with their city of origin.

After dividing subjects into groups of Mumbaikars and Bangaloreans, subjects played a quiz in which group performance paid off to strengthen the feeling of belonging to a group.

#### Economic heterogeneity

Economic heterogeneity is induced by varying the endowment *E* that players receive at the start of each period. Under economic homogeneity, all appropriators receive *E* = 50 to invest in appropriation. Under economic heterogeneity, however, two players receive *E* = 40 and two players receive *E* = 60 (see i.a. Cherry et al. [4] for a similar operationalisation of economic heterogeneity based on variations in endowment). The total endowment of the group is 200 for all groups in all treatments.

#### Four combinations

The four treatments that are applied in the experiment are (1) economic heterogeneity [EH], (2) sociocultural heterogeneity [SH], (3) economic and sociocultural heterogeneity [EHSH], and the control treatment (4) no heterogeneity [NH] (The IND study here following Van Klingeren [31]). Note that in the EHSH treatment, economic heterogeneity is lined up with sociocultural heterogeneity; that is, two members of the same group receive *E* = 60 and the two other members, who are both members of the other group, receive *E* = 40. In the EH treatment, two randomly chosen players receive *E* = 40 and the other two receive *E* = 60. Table 1 shows the treatments and the number of subjects in each treatment for the UKNL and IND studies. In the treatments without sociocultural heterogeneity—EH and NH—there is an unequal division of Mumbaikars and Bangaloreans due to the difficulty of recruitment of Bangaloreans, who were a minority at the FLAME University campus. However, a two-level multilevel regression on resource size with random effects for groups and a three-level multilevel regression on appropriation with random effects for groups and subjects show that being a Mumbaikar or Bangalorean does not significantly affect behaviour on respectively the macro-level (B = −0.180, p = 0.971) or the micro-level (B = 1.828, p = 0.141). For these treatments, the unequal division of Mumbaikars and Bangaloreans is thus no issue. For all treatments it is the case that subjects know their own endowment and the endowment of others in their group; just as they know their own group identity and the group identity of others in their group. They see all this information in a box on the screen every period.

**Table 1:** Overview of treatments.

	Treatment	Operationalisation	UKNL	IND Mumbai	Bangalore
1. EH	Economic heterogeneity Sociocultural homogeneity	Different endowments Same painter / city identity	N = 92	N = 24	N = 12
2. SH	Economic homogeneity Sociocultural heterogeneity	Same endowments Different painter / city identity	N = 88	N = 18	N = 18
3. EHSH	Economic heterogeneity Sociocultural heterogeneity	Different endowments Different painter / city identity	N = 84	N = 18	N = 18
4. NH	Economic homogeneity Sociocultural homogeneity	Same endowments Same painter / city identity	N = 80	N = 28	N = 8

#### Analytical strategy

To investigate statistical significance of the differences between treatments, two-level multilevel regression models on the macro-level outcome resource size with random intercepts for groups, and three-level multilevel regression models on the micro-level outcome appropriation effort with random intercepts for groups and subjects are conducted. The results can be found in the tables of S2 Table. Information on the modelling-decisions such as control variables can be found there too. See Van Klingeren [31] for a more detailed description regarding the formal regression functions and modelling decisions for the multilevel models. The main results from the multilevel regression models are illustrated and discussed using predictive plots in the Results section.

### Player type classification

After the respondents play the Fishing Game in the experiment, a player type can be constructed based on their individually displayed behaviour. Based on research using cooperative type classification, the expectation is that most subjects will be free-riders, conditional cooperators or neutral cooperators, with conditional cooperators being the largest group [38, 50–56]. To explain the differences in behaviour found in the two CPR experiments, an LCP score is calculated for each subject following Kurzban & Houser [38], to classify them as free-rider, conditional cooperator or neutral cooperator.

#### LCP classification process

The LCP score of subjects is calculated as the estimated parameters of a multilevel regression of the subject’s appropriation effort on the mean appropriation effort of others in the previous period, with a random effect and random intercept on the subject level. The combined data of UKNL (for the first 30 periods) and IND studies is used in this model. This approach is based on Kurzban & Houser [38] who use a similar strategy using OLS regression. In the current strategy, however, the multilevel regression also includes individual characteristics such as level of general trust from the IG, age, sex, number of acquaintances in the experimental session and experience with game theory. Using a sum-to-zero constraint, the model also controls for the country that the experimental session took place in. The latter makes sure that the model controls for any cultural traits that may systematically influence the dependent variable. The outcome of this model provides an intercept and a slope for each subject, which indicates a subject’s baseline willingness to cooperate and the subject’s responsiveness to the appropriation of others respectively.

To add some uncertainty in the LCP scoring process—and thus robustness in the process—the following strategy is used. First, after calculating the slope and intercept as part of each subjects’ LCP score, two simulation samples are created with the number of simulations *N*_*s*_ = 100, based on two random normal distributions: one with the individual slope as its mean and the standard deviation of the slope from the regression model as its standard deviation, and the other with the individual intercept as its mean, and the standard deviation of the intercept from the regression model as its standard deviation. This gives us 100 simulations for the slope and 100 simulations for the intercept for each subject.

Using these simulations of LCP scores, subjects are divided into types. Firstly, the LCP scores are thought of as dots on a grid with intercept *α* on the y-axis and slope *β* on the x-axis. This grid is divided on an intercept threshold $\tau_{s}^{\alpha }$ where *α* indicates the intercept and *s* connotes the simulation aspect. Secondly, the grid is divided on a slope threshold $\tau_{s}^{\beta }$ where *β* connotes the slope.

The two thresholds $\tau_{s}^{\alpha }$ and $\tau_{s}^{\beta }$ divide the grid into four quarters. The top two quarters are free-riders, who on average invest more than the cooperative threshold of 30 in appropriation. The bottom-right quarter are conditional cooperators, who invest cooperatively when others in their group do so as well as indicated by an intercept equal to or lesser than 30 and a slope above 0. A slope below 0 would also be conditional. However, conditional cooperators are defined as players that cooperate when others do so too, and whenever others over-exploit the resource, conditional cooperators do so too. A negative slope would mean that the subjects react to cooperation with non-cooperation and vice versa, which is not coherent with the present conceptualisation of conditional cooperators. Hence, the slope sign must be positive to fit the conditional cooperative type.

The bottom-left quarter includes subjects of whom the basic appropriation is below the cooperative threshold, and who invest more when others invest less and vice versa. As there is no player type in behavioural theory that describes this kind of behaviour—and there is thus no expectation of many subjects falling into this category—we will call the subjects in this group the residual subjects.

These three classifications, however, do not cover the unconditional or neutral cooperative player, who invests 30, close to 30, or lower in each period, regardless of what others do. All subjects know that 30 is the cooperative threshold of appropriation as this is explained and illustrated in the instructions, so we can expect a decent percentage of subjects to follow that strategy over the entire game. To classify this player type, some uncertainty is added into the thresholds. A normally distributed simulation sample is created for the intercept and the slope values, with the hard thresholds of $\tau_{s}^{\alpha }=30$ and $\tau_{s}^{\beta }=0$ as means, and standard deviations of respectively *sd*_*τα*_ = 2 and *sd*_*τβ*_ = 0.1. The standard deviations for $\tau_{s}^{\alpha }$ and $\tau_{s}^{\beta }$ were chosen to be 2 and 0.1 so that players who invest very close to 30 each period—albeit with some variation, as that is inevitable with real human players—are selected as neutral cooperators. Smaller standard deviations could result in neutral cooperators being wrongfully classified as other types, whereas larger standard deviations could lead to other type players being wrongfully classified as neutral cooperators as the margin would be too big.

The setting of the standard deviations is arbitrary to some degree. However, as can be seen from the results section later on, the classification results—despite the added uncertainty in LCP score simulations for subjects and classification threshold simulations—do match type percentages that are often found in the literature, which provides confidence in the chosen classification rules.

**Fig 1:**
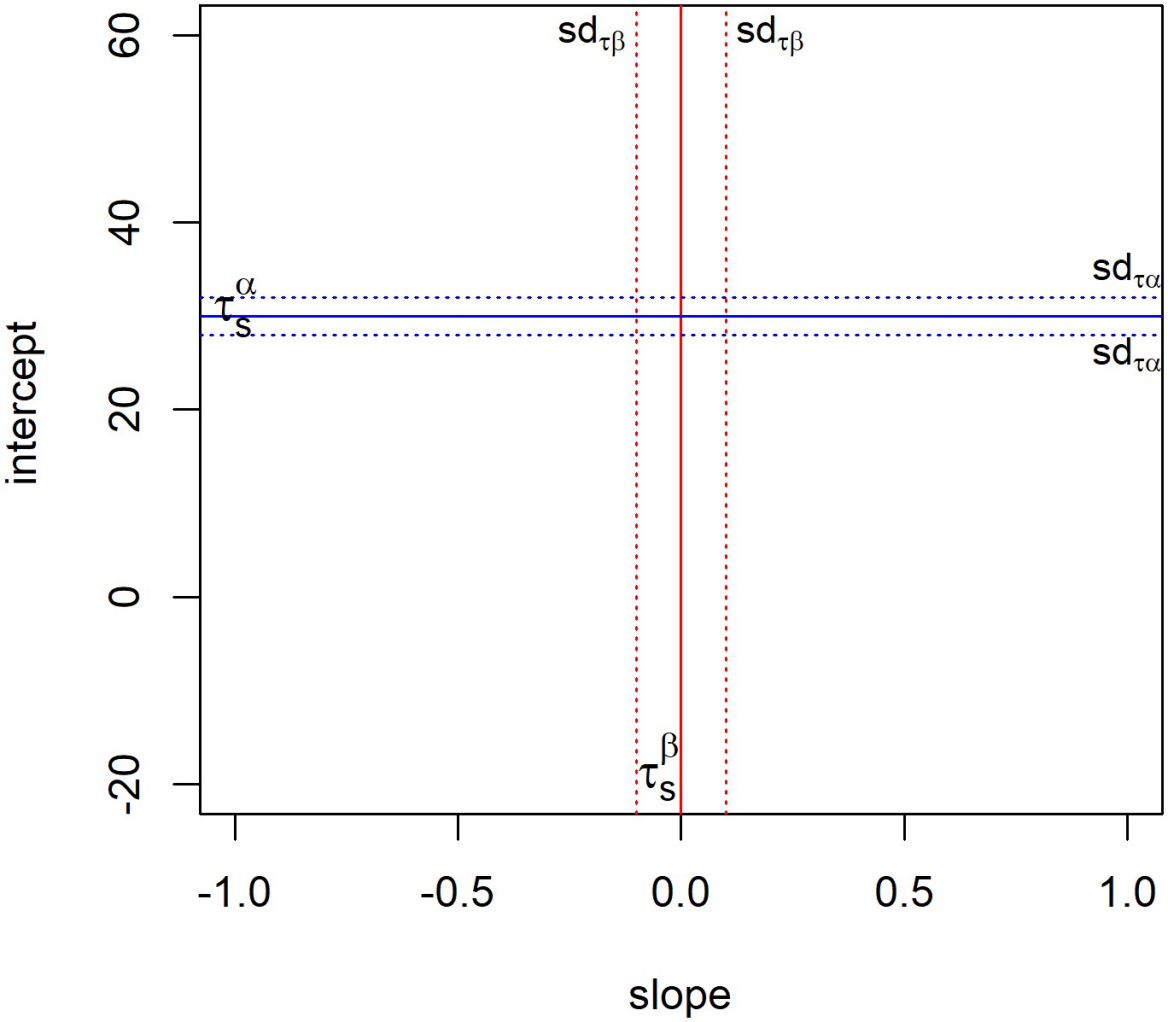
Classification grid.

A visual representation of the classification grid as just described is presented in Fig 1.

The actual classification happens by comparing each of the 100 simulated intercepts and slopes for each individual with each simulated threshold for the intercept and slope. This way, subjects get a probability score to be a free-rider, conditional cooperator, or residual subject. Once a subject has a positive probability for all three types, the subject is classified as a neutral cooperator—the LCP of these subjects lies around the *α* = 30 and *β* = 0 values, and is thus a neutral cooperator. As this description does not capture the neutral cooperative players who invest much less than the threshold of 30, another classification rule is added for the neutral cooperators: investing equal to or less than the cooperative threshold, and having a non-zero probability of being a residual subject or a conditional cooperator. The latter part of the argument is a way of specifying that the slope of these players should be close to 0 (implying unconditional behaviour). To summarise, Table 2 shows the formal rules for player type classification:

**Table 2:** LCP type classification rules.

Type	Rule
Free-riders [FR]	$1(\alpha_{s}>\tau_{s}^{\alpha })$
Conditional Cooperators [CC]	$1(\beta_{s}>\tau_{s}^{\beta }\;\&\;\alpha_{s}\leq \tau_{s}^{\alpha })$
Residual Subjects [RS]	$1(\beta_{s}<\tau_{s}^{\beta }\;\&\;\alpha_{s}\leq \tau_{s}^{\alpha })$
Neutral Cooperators [NC]	$\sum_{s=1}^{N_{s}}1(\alpha_{s}\geq \tau_{s}^{\alpha })>0\;\&$
	$\sum_{s=1}^{N_{s}}1(\beta_{s}>\tau_{s}^{\beta }\;\&\;\alpha_{s}\leq \tau_{s}^{\alpha })>0\;\&$
	$\sum_{s=1}^{N_{s}}1(\beta_{s}<\tau_{s}^{\beta }\;\&\;\alpha_{s}\leq \tau_{s}^{\alpha })>0$
	*or*
	$1(\alpha_{s}\leq \tau_{s}^{\alpha })\;\&$
	$\sum_{s=1}^{N_{s}}1(\beta_{s}>\tau_{s}^{\beta }\;\&\;\alpha_{s}\leq \tau_{s}^{\alpha })>0\;\&$
	$\sum_{s=1}^{N_{s}}1(\beta_{s}<\tau_{s}^{\beta }\;\&\;\alpha_{s}\leq \tau_{s}^{\alpha })>0$

### Agent-based models

**Fig 2:**
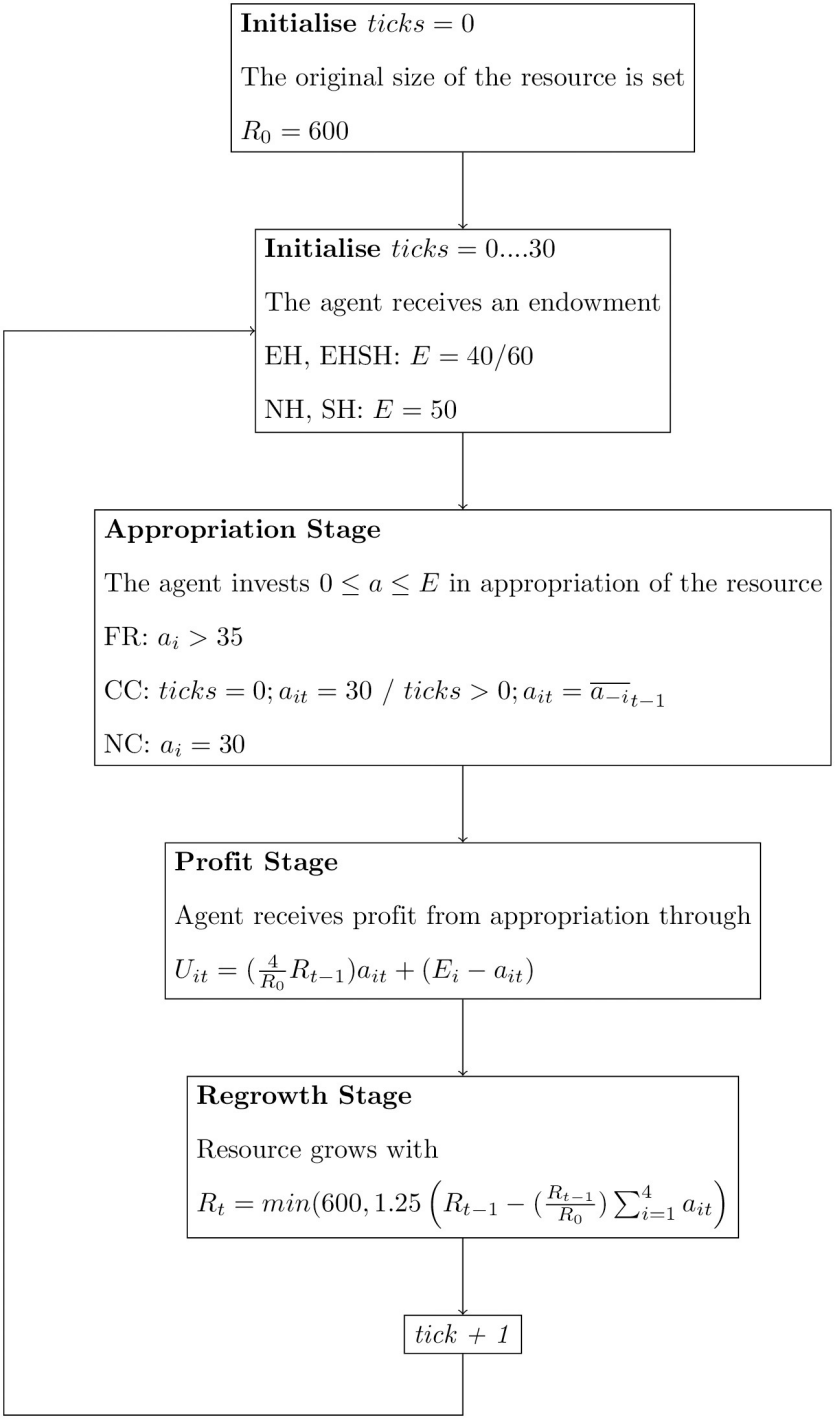
A flow chart overview of the mixed ABM model as coded in NetLogo [57].

After calculating the percentages of free-riders, conditional cooperators and neutral cooperators in each experimental treatment, the player type distributions are used to simulate the experimental results with ABMs. ABMs will translate information from the player type classification to a more basic understanding of cooperation by using simple theoretical agents to replicate complex behaviour. The ABM game that the agents “play” is a direct copy of the Fishing Game as played in both experiments, with the exception that no sociocultural heterogeneity was introduced to the agents. Agents would not act differently by being placed in groups that only differ by name, and do not have rules on how to behave differently from the other group. On top of that, it was not needed for agents to be grouped as the purpose of the ABMs was to fit agents’ behaviour to experimental outcomes according to player type distributions found in each treatment, which in itself were already affected by the treatment effects. For a schematic overview of the mixed model ABM procedure in NetLogo [57] see Fig 2.

For the ABMs, simple agents based on the free-rider, conditional cooperator and neutral cooperator are used. In addition, a random agent model is added as baseline for comparison. The free-riding agent will never cooperate. In the Fishing Game, this means that the free-riding agent will never appropriate *a* = 30 or close to *a* = 30 and will always invest between *a* = 35 and *a* = *E* in appropriation. The free-riding agent appropriates the same amount every period. The cooperative agent will always invest 30 in appropriation, as this is the cooperative threshold to invest when the resource size is at its full size, and will never contribute to a decrease in resource size. Although the cooperative agent could also invest much less than 30 and still be strictly cooperative, this is not a behaviour that is very realistic based on the behaviour found in the experiments. Hence, a cooperative player is modelled as investing 30 units in appropriation. In addition, the goal of the ABM exercise is to replicate complicated outcomes with simple agents, with simple rules. The conditional cooperative agent will only cooperate if other players cooperate as well [51, 58]. In the Fishing Game, conditional cooperators appropriate *a* = 30 in the first period, and will then appropriate as much as the mean appropriation of others in the previous period. Lastly, the random agent is a baseline agent to compare the other agents with, that does not have a specific strategy. Each period, they will invest a random amount of *a* units in appropriation of the resource, in the range of [0, *E*].

The ABMs that will be used to fit the data comprise a mixed model with free-riders, conditional cooperators and neutral cooperators—informed by player type distributions found in the experimental data—and single agent models with only one of the agents present. To fit the EH and EHSH data, two agents will be assigned *E* = 40 and two agents will be assigned *E* = 60.

#### Calculating the fit

How well a model matches the experimental data is measured with a fit score. The fit *f* of a behavioural model under a certain set of parameters is calculated by taking the difference between values of key variables in the simulated data and the data from the UKNL and IND studies. 5000 simulation games are run using NetLogo [57], with parameters of the mixed model set to match proportions of player types as found by the LCP classifications in the experimental data. The simulated data is then compared to the experimental data to create a fit score. The four metrics that will be measured and compared are (1) resource size, (2) appropriation effort, (3) change in appropriation, and (4) individual profit. Resource size is a macro-level outcome, whereas the other variables are micro-level outcomes. The average value of these variables per period per treatment from the experimental data are compared to the value of these variables in each simulation of the Fishing Game. The fit will be calculated using the following function following Janssen & Baggio [39]:

$$\begin{array}{c} f=\prod_{m=1}^{4}(1-\sqrt{\frac{\sum_{t=1}^{n_{mt}}{\left(s_{mt}-d_{mt}\right)}^{2}}{n_{mt}}}/\text{max}\left(d_{m}\right)) \end{array}$$

where *m* is the metric, *t* is the period, *d*_*mt*_ is the average observed data and *s*_*mt*_ is the average simulated data over the 5000 simulations for *n*_*mt*_ observations or periods. The final fit score *f* is the product of the fit score of the four variables. Taking the product of the fit score per metric, which will be between 0 and 1, ensures a more conservative fit score than taking for instance the sum by enlarging the difference between a good and a bad fit. The calculation of *f* does not control for the number of parameters. To check the robustness of *f* while controlling for the number of parameters in the ABM the adjusted R^2^ was calculated for each metric, treatment and study and can be found in S3 Table. Regarding the relative fit between the models, the adjusted R^2^ gives the same conclusion as *f*. Although the adjusted R^2^ is not the most appropriate way of calculating the fit between so few data points and simulations (as we are only comparing the average outcomes over 30 periods) it does support the rank order of model fit amongst the ABMs.

For the EH and EHSH treatments the fit score *f* and the adjusted R^2^ for micro-level metrics appropriation effort, change in appropriation, and profit are measured and compared separately for high and low endowed players, to make sure the simulations take into account the specific behaviours of subjects with high and low endowments. After calculating the fit score *f* and the adjusted R^2^ for these metrics separately for high (*E* = 60) and low (*E* = 40) endowed players, the average *f* and adjusted R^2^ of high and low endowed players is taken to represent the final score for that particular metric.

## Results

### Experimental results

**Fig 3:**
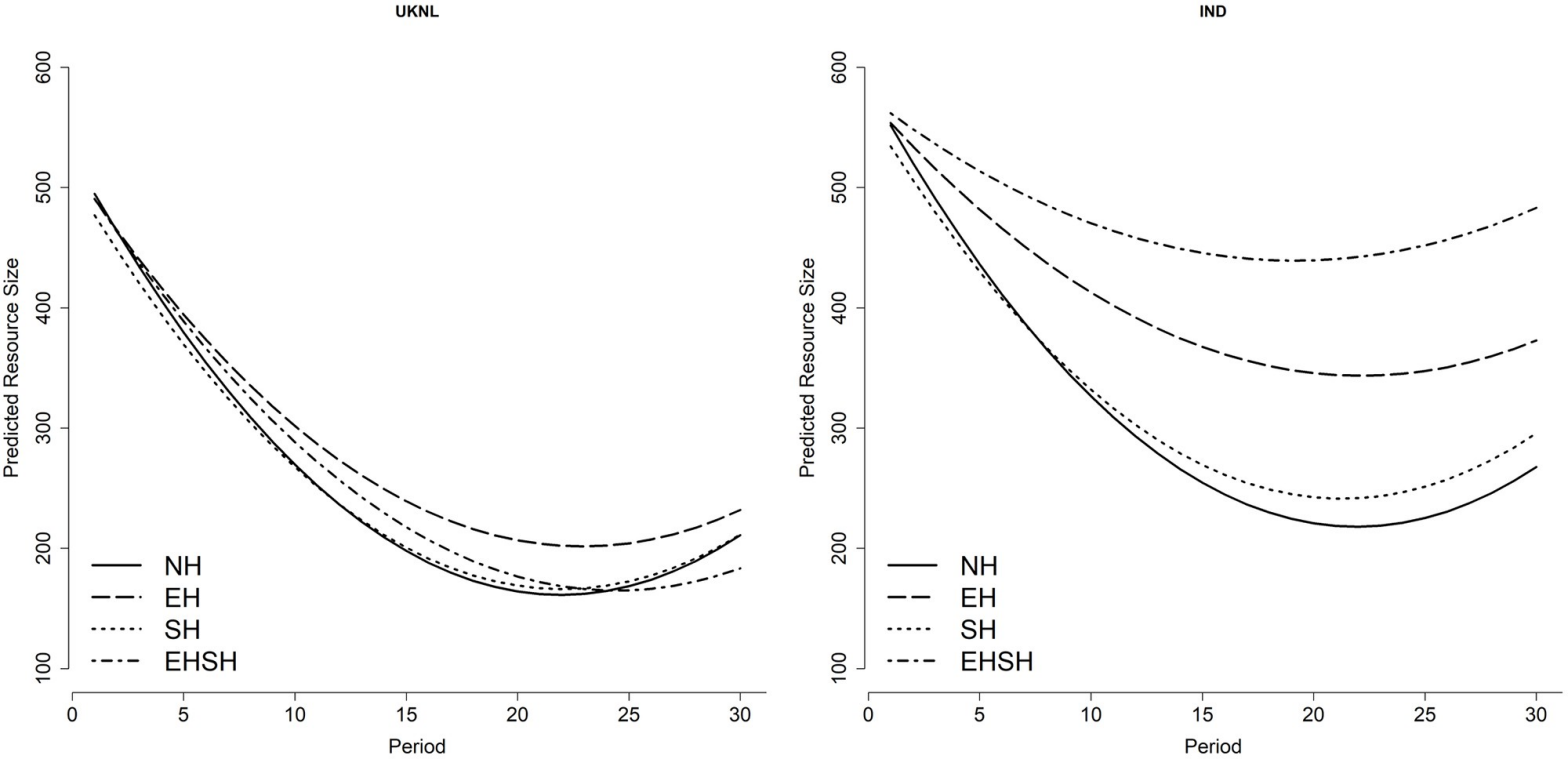
Predicted resource size per treatment per period.

Fig 3 shows the predicted resource size based on the two-level multilevel regression coefficients of the main, interaction and quadratic interaction treatment effects over time reported in model 3 of S2 Table and serves as a visualisation of the results. In the UKNL study it is visible that the EH treatment performs better over time relative to the NH treatment—this effect is significant. The SH treatment too performs significantly better per period than NH in the UKNL study, however with a smaller effect size. The combination treatment EHSH has a significantly higher resource size over time relative to NH for the UKNL treatment—although decreaslingly so, which results in EHSH having the lowest resource size of all treatments by period 25. When looking at the effects for the IND study, the results show that the EH treatment does even better relative to the NH treatment than EH in the UKNL study. The biggest change in the IND study compared to the UKNL study is the effect per period of the EHSH treatment relative to the NH treatment, which is positive and very substantial.

**Fig 4:**
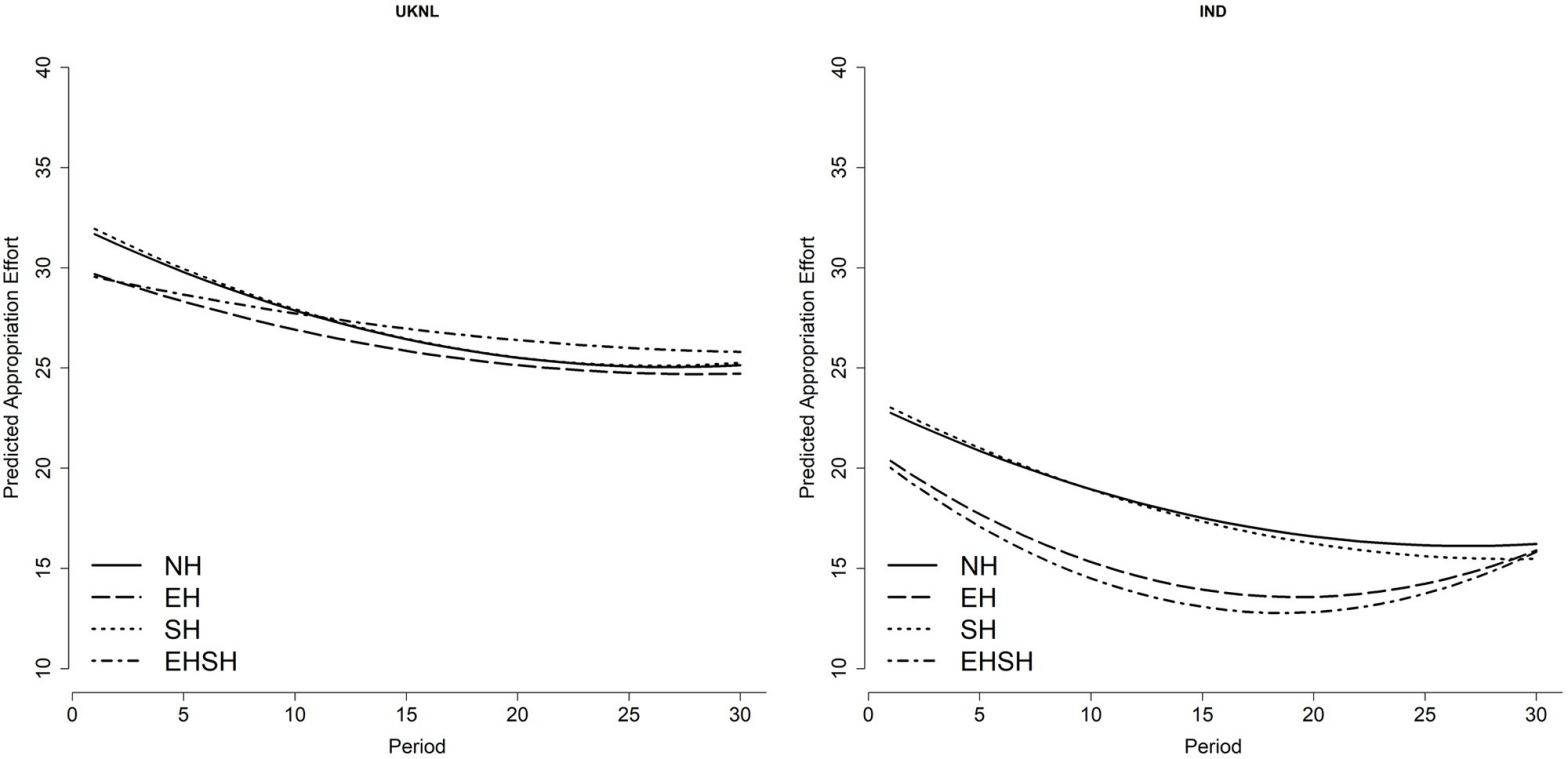
Predicted appropriation effort per treatment per period.

Fig 4 visualises the coefficients of the main, interaction and quadratic interaction treatment effects over time of the three-level multilevel regression model on appropriation effort (model 3, table provided in S2 Table). It shows that the EHSH treatment has a significantly higher appropriation effort per period relative to NH in the UKNL treatment, which decreases over time. For the IND study, the opposite is visible: appropriation per period in the EHSH treatment is significantly lower than in the NH treatment. It is clear from the graphs that appropriation in the SH and NH treatments are very close to each other in both the IND and UKNL study. Although the difference is not statistically significant, in both the IND and UKNL study subjects in the EH treatment appropriates less than the NH and SH treatments. But whereas in the UKNL study the EHSH treatment has the highest appropriation effort from period 10 on, this treatment has the lowest appropriation effort in the IND study for the entire duration of the game.

It is remarkable that the EHSH treatment performed so differently in the UKNL and the IND study, especially since the behaviour in the other three treatments is very similar between the two studies. The behaviour of subjects in the EH treatment in the IND study supports results from Van Klingeren [31] with regard to the positive effect of economic heterogeneity on cooperation relative to homogeneity. The results of the EHSH treatment, however, contradict the result that the combination of economic and sociocultural heterogeneity has a negative effect on cooperation.

#### Trust and fairness

Although the multilevel regressions on the macro- and micro-level show significant positive effects of average general trust in the group and individual general trust on cooperation, ordinal logistic regression models on trust in other players and subjective trustworthiness of others show that subjects’ final profit rather than treatment significantly affects how subjects answered the post-experimental survey questions on trust in the Fishing Game in the UKNL study and partially in the IND study. The ordinal logistic regression tables can be found in S4 Table.

Another variable that was measured in the post-experimental survey—and which may provide some additional insights on why subjects cooperated or not—is fairness. Regarding fairness of the division of endowments, no notable differences are found between the UKNL and IND studies. But, whereas the EHSH subjects in the UKNL study report significantly lower on the feeling of being treated fairly (OR = 0.44, p = 0.003), this is not the case for the IND study, where only the EH treatment reports marginally lower on the feeling of being treated fairly (OR = 0.41, p = 0.053). In the UKNL study, the division of higher and lower endowments by Klee or Kandinsky preference thus significantly impacted whether subjects felt treated unfairly or not during the game for the EHSH treatment subjects, while this was not the case for the division by city origin. This can be a reason for EHSH subjects to behave more cooperatively in the IND study, which would explain the difference in EHSH outcomes between the UKNL and IND studies.

### Player type results

Now the significant differences in behaviour are detected, player type classification using LCP scores will be used to translate the found behaviour into free-riding, conditional cooperative and neutral cooperative categories. This will offer a different understanding of the statistical outcomes by providing insights on a more granular level; the level of the individual player.

**Fig 5:**
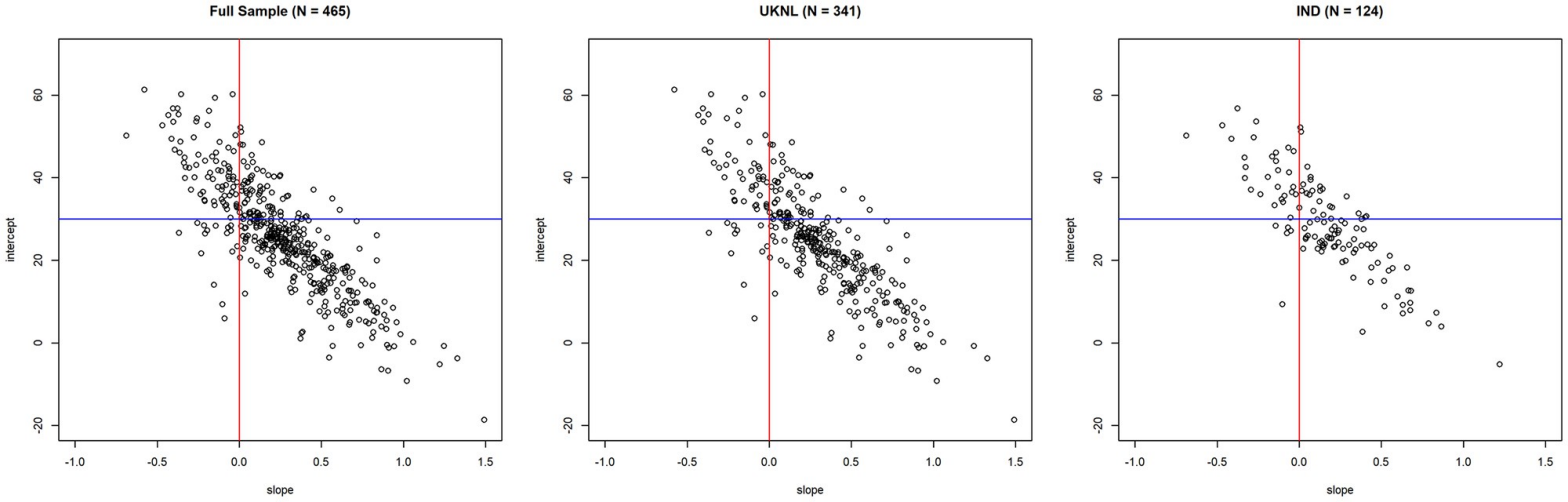
Plot of intercept and slope of LCP scores.

The LCP classification rules are visually represented in the division of the grid in Fig 5, which shows a dot chart of LCP scores of subjects for the combined data and per country. Note that the sample size is decreased, as there are some missing observations on the post-experimental survey which includes questions on individual characteristics that were used as control variables in the LCP calculation. The horizontal blue line shows the average threshold for the intercept, and the vertical red line shows the average threshold for the slope.

As is visible, the LCP scores are distributed similarly for the UKNL and IND studies. The plot shows that intercept and slope are negatively correlated. This makes sense, as it is not likely for subjects that already have a very uncooperative baseline appropriation (slope) to be positively affected by others in their group. Vice versa, subjects with a lower baseline appropriation are more likely to be conditionally cooperative and thus be affected by what others in their group do.

**Fig 6:**
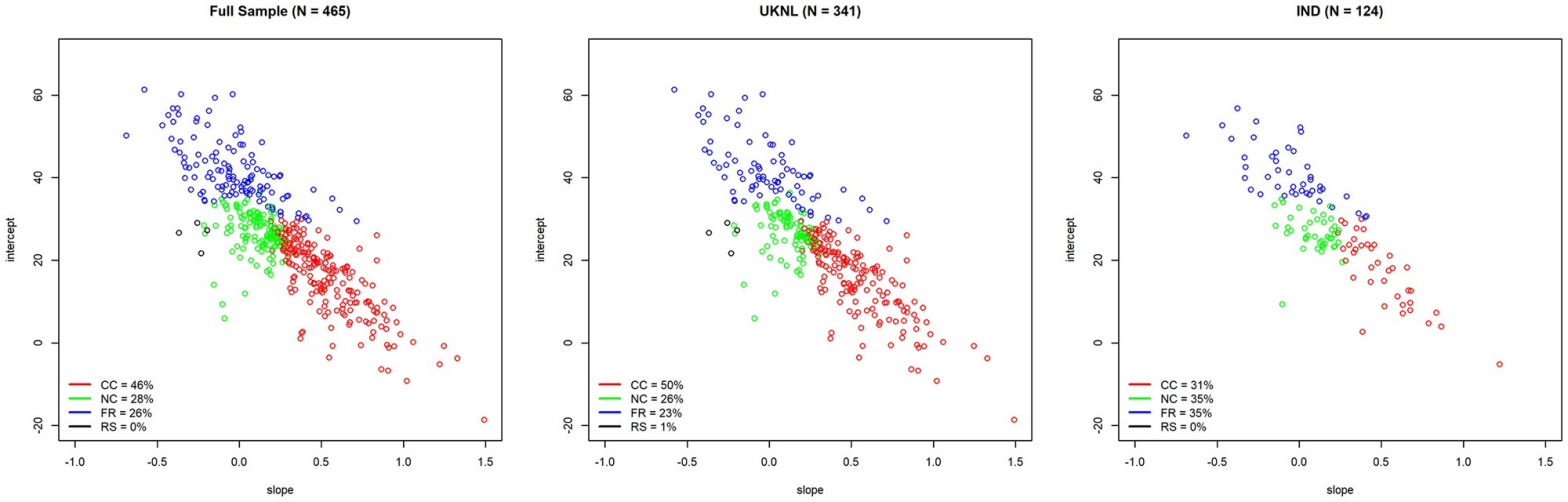
Plot of intercept and slope of LCP scores—Types by colour.

Fig 6 shows the division of subjects over the four defined player types, including the rounded up percentages of each type in the sample, with the conditional cooperators presented in red, the neutral cooperators in green, the free-riders in blue and the residual subjects in black.

The percentages of types found in the experimental samples—50% (UKNL) and 31% (IND) conditional cooperators, 26% (UKNL) and 35% (IND) neutral cooperators and 23% (UKNL) and 35% (IND) free-riders—match the percentages found in other research on cooperatives types such as Fishbacher, Gächter & Fehr [51] who find 50% conditional cooperators and 30% free-riders, and Kurzban & Houser [38] who find 63% conditional cooperators—or reciprocators, as they label them -, 13% cooperators and 20% free-riders. Other research reports generally between 45% and 80% conditional cooperators [52–55]. The percentage of conditional cooperators in the IND sample is slightly lower than expected with only 31%, as conditional cooperators are usually the largest player-type group. However, as Kocher et al. [55] show in a comparative study of player types in Austria, the United States and Japan, the distribution of player types and especially the extent of conditional cooperation differ per country. As expected, only very few subjects fall in the residual category.

**Fig 7:**
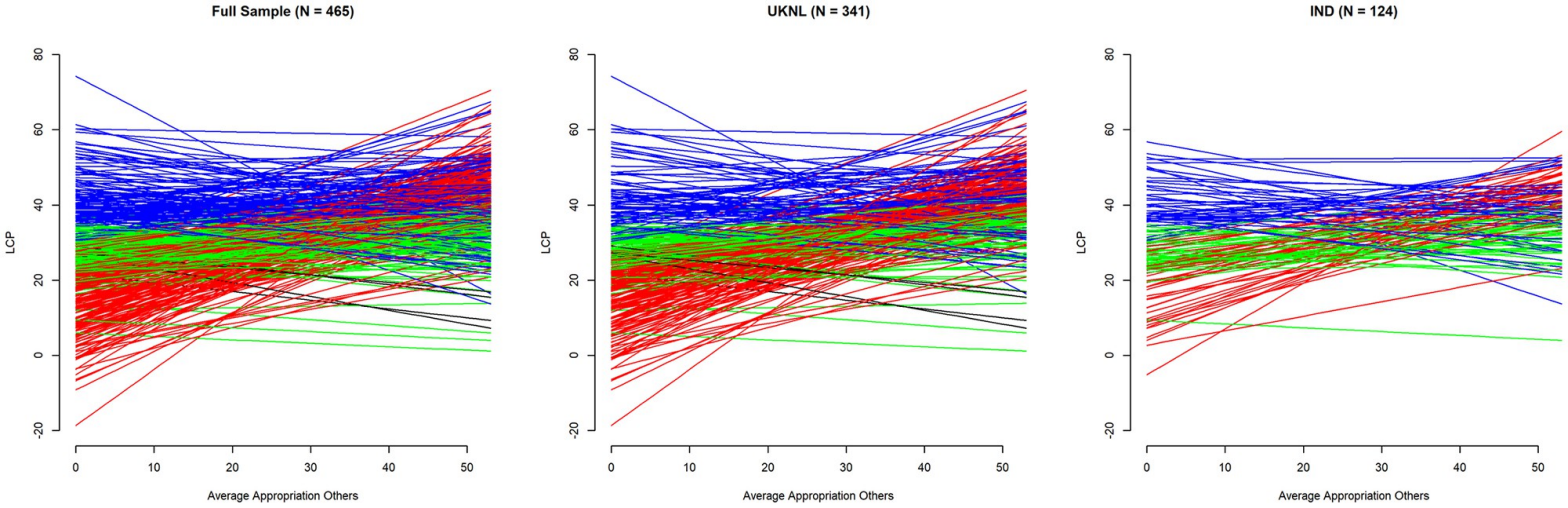
LCP (intercept & slope) by average appropriation of others (t-1)—types by colour.

To get a more concrete idea of what each subject would *do* given a specific amount of appropriation by the other players according to their LCP score, Fig 7 provides a line graph of subjects’ LCP scores over the mean appropriation of other players in the previous period.

**Fig 8:**
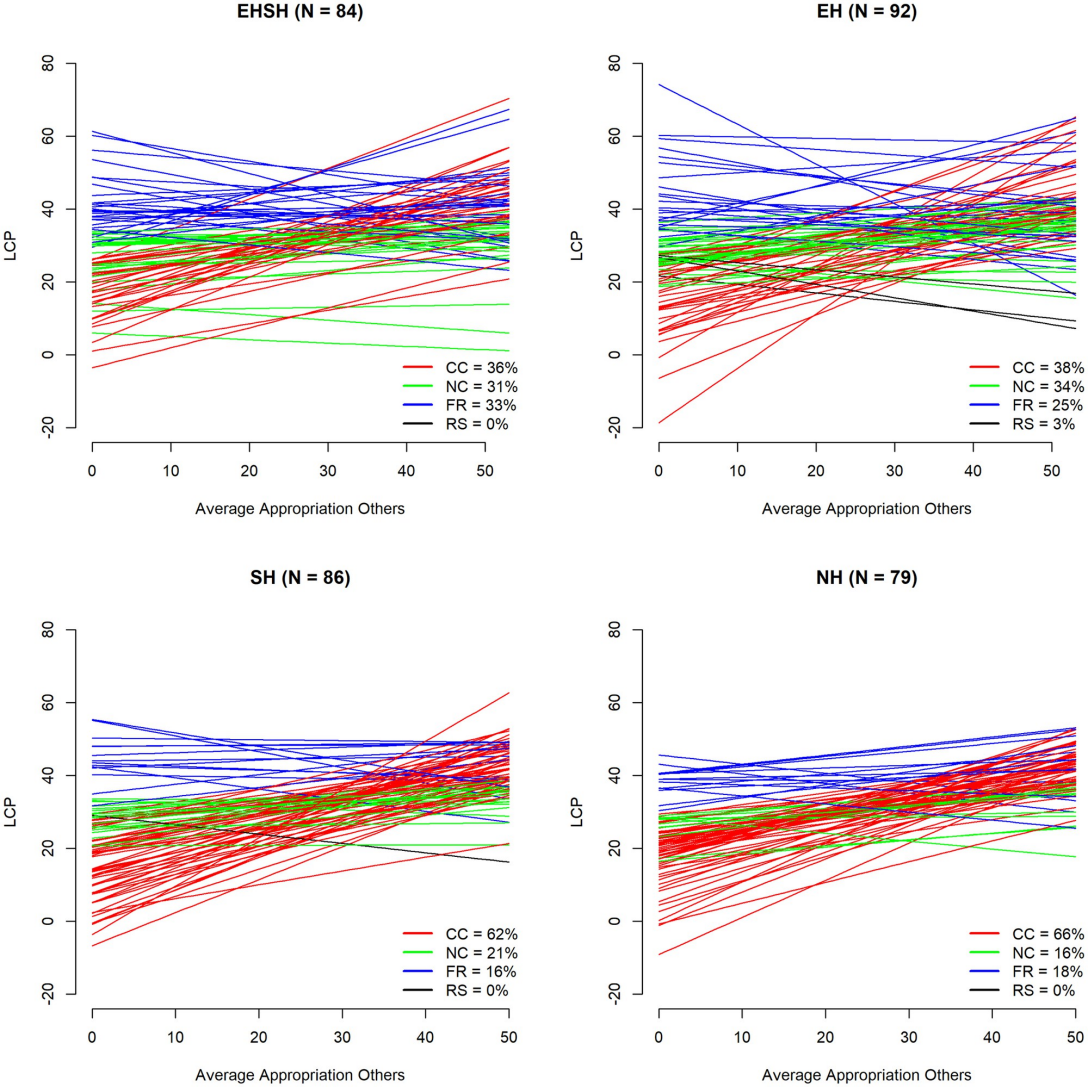
LCP (intercept & slope) by average appropriation of others (t-1) by treatment UKNL.

**Fig 9:**
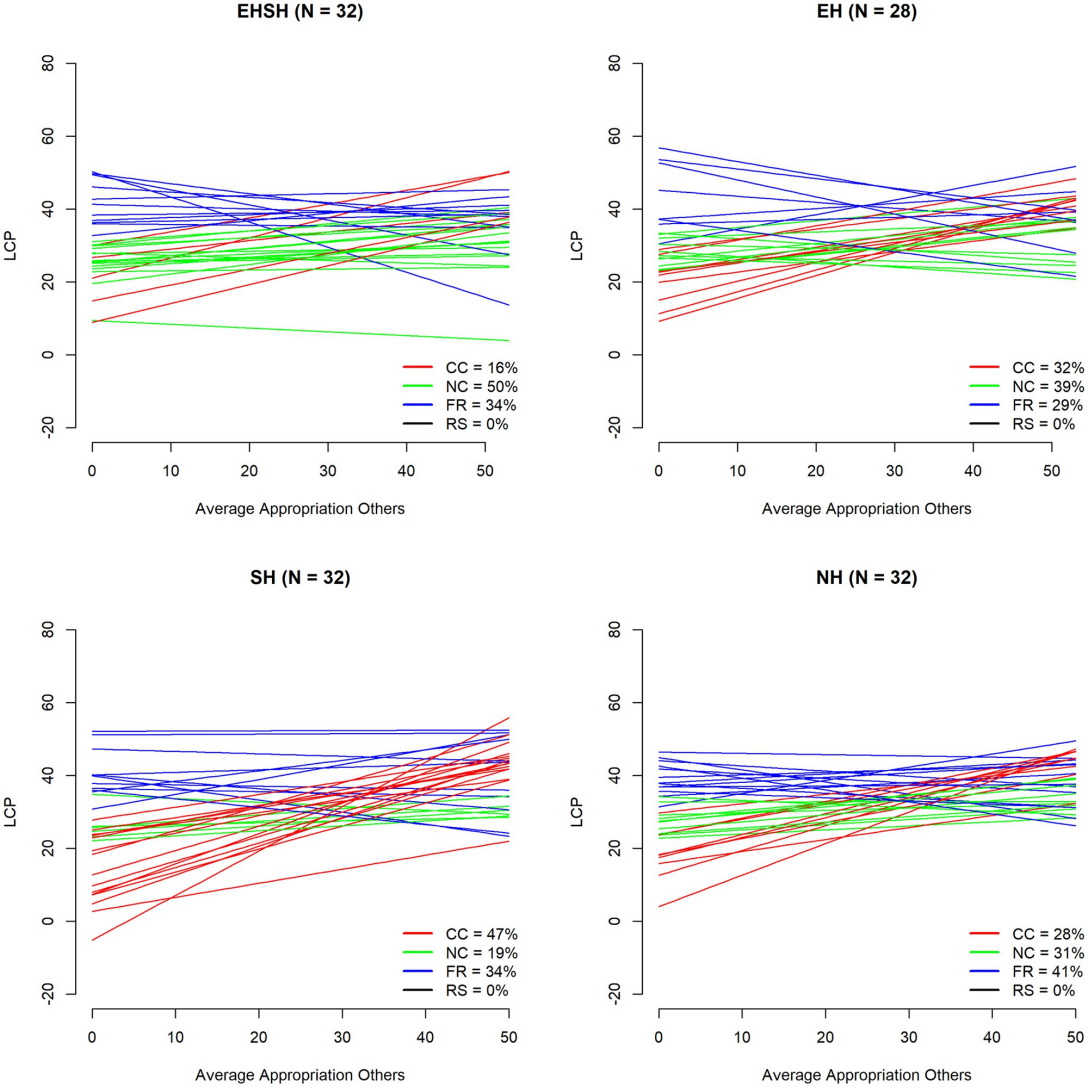
LCP (intercept & slope) by average appropriation of others (t-1) by treatment IND.

One explanation for the differences in behaviour between treatments in the UKNL and IND studies could be a different division of player types over treatments. Figs 8 and 9 show the division of player types over the four treatments in the UKNL sample and the IND sample respectively.

Fig 8 shows that the EHSH treatment in the UKNL study has the highest percentage of free-riders (33%), which is part of the reason that the EHSH treatment performs worst in the long run. The EH treatment has the highest percentage of neutral cooperators, which is part of the reason that the EH treatment did better than the other treatments, despite having a higher percentage of free-riders than the SH and NH treatments: whereas a conditional cooperators would increase their appropriation as a reply to the increased average appropriation of others, neutral cooperators keep investing around the cooperative threshold, which keeps the resource size up. Fig 9 shows that the EHSH treatment in the IND study has the highest percentage of neutral cooperators which, as just described, can explain the success of this treatment. The EHSH treatment also has a low percentage (16%) of conditional cooperators compared to all other treatments in the IND and UKNL studies, which means that there is less of a reaction to free-rider behaviour and thus the resource size will stay relatively high.

### ABM results

It is now apparent that economic and sociocultural heterogeneity affect player type distribution. The results from the ABM simulations will show whether simple free-riding, conditional cooperative or neutral cooperative agents in ABM simulation models can replicate the experimental outcomes on the macro- and micro-level.

Table 3 shows the fit score *f* of the mixed and single ABMs for each of the four metrics that describe the macro- and micro-level data. The mixed models consist of free-riders, conditional cooperators and neutral cooperators according to the percentages found in the LCP classification as represented in Figs 8 and 9. Note that the residual subject category is not represented in the ABMs. The type designation in the ABMs is based on the probability to be a conditional cooperator and the probability to be a free-rider—if the agent is neither they are neutral cooperators. The percentage of residual subjects is thus added to the neutral cooperation category. As this concerns only a couple of players this makes no substantive difference for the interpretation of the ABM results.

**Table 3:** Fit score *f* per ABM per treatment.

		UKNL EH	SH	EHSH	NH	IND EH	SH	EHSH	NH
Mixed Model*									
	General Fit	0.724	0.699	0.751	0.728	0.705	0.693	0.578	0.705
	General Fit *E* = 60	0.812		0.791		0.775		0.605	
	General Fit *E* = 40	0.640		0.709		0.637		0.550	
	Fit Resource Size	0.944	0.859	0.947	0.893	0.933	0.871	0.763	0.900
	Fit Profit	0.889	0.912	0.954	0.933	0.895	0.917	0.861	0.938
	Fit Appropriation	0.900	0.920	0.880	0.910	0.900	0.901	0.912	0.890
	Fit Change Appropriation	0.963	0.974	0.943	0.964	0.938	0.963	0.964	0.939
Cooperative									
	General Fit	0.391	0.387	0.380	0.376	0.425	0.438	0.684	0.381
	General Fit *E* = 60	0.441		0.394		0.474		0.712	
	General Fit *E* = 40	0.337		0.364		0.373		0.656	
	Fit Resource Size	0.626	0.578	0.599	0.571	0.677	0.624	0.841	0.585
	Fit Profit	0.713	0.757	0.756	0.753	0.740	0.790	0.901	0.766
	Fit Appropriation	0.910	0.910	0.890	0.910	0.906	0.924	0.936	0.906
	Fit Change Appropriation	0.963	0.974	0.943	0.964	0.938	0.963	0.964	0.939
Free-rider									
	General Fit	0.375	0.413	0.384	0.415	0.319	0.365	0.193	0.383
	General Fit *E* = 60	0.379		0.403		0.328		0.205	
	General Fit *E* = 40	0.371		0.365		0.310		0.181	
	Fit Resource Size	0.630	0.671	0.658	0.678	0.574	0.620	0.409	0.656
	Fit Profit	0.781	0.789	0.777	0.794	0.746	0.755	0.626	0.777
	Fit Appropriation	0.790	0.800	0.800	0.800	0.794	0.810	0.780	0.801
	Fit Change Appropriation	0.963	0.974	0.943	0.964	0.938	0.963	0.964	0.939
Random									
	General Fit	0.368	0.392	0.397	0.381	0.401	0.434	0.651	0.382
	General Fit *E* = 60	0.453		0.406		0.484		0.722	
	General Fit *E* = 40	0.290		0.386		0.324		0.581	
	Fit Resource Size	0.640	0.592	0.614	0.585	0.691	0.637	0.855	0.598
	Fit Profit	0.729	0.830	0.843	0.826	0.754	0.858	0.932	0.833
	Fit Appropriation	0.820	0.820	0.810	0.820	0.822	0.826	0.848	0.819
	Fit Change Appropriation	0.964	0.975	0.943	0.964	0.937	0.963	0.964	0.938

* This model consists of cooperative agents, conditionally cooperative agents and free-riding agents according to the player type proportions found per treatment in the LCP score calculation as apparent from Figs 8 and 9.

The single agents and random agent models were added to be able to compare the more complex model with simpler baseline models. For all but one treatment—EHSH in the IND study—the fit of the mixed model is higher than the simple models, indicating that the mixed model is more successful in replicating outcomes similar to the experimental data. This is different for the EHSH treatment of the IND study, of which the fit score is higher in the random and the cooperative models. This is not surprising, as the shape of the curve describing resource size and appropriation effort over time for this treatment differs from the other treatments in the IND and UKNL studies.

Whereas the mixed model does well in predicting most treatments’ resource size curves that follow the pattern of a quick decrease in the first 10 periods followed by a flattening of the curve—as we also see in other CPR experiments [59, 60]—it does not do well in predicting a resource size curve as visible in the EHSH treatment of the IND study. The fact that the random model has a relatively high fit for the EHSH treatment in the IND study does not necessarily mean that the EHSH subjects acted randomly. The random agent appropriates on average 30, which is the same for the cooperative agent. Both single agent models thus have similar outcomes and a similar fit. However, when looking at the appropriation behaviour, it is visible that the cooperative model has a higher fit for general fitness for player with a low endowment and for the appropriation metric. It thus performs better than the random ABM on important aspects.

Regarding the fit of the mixed model with the macro- and micro-level outcomes, Table 3 shows that overall the macro-level variable resource size is especially well replicated in the mixed model compared to the single agent models. The micro-level variable profit too has a consistently higher fit score in the mixed model compared to the single models. Average appropriation is replicated slightly better in the cooperative model compared to the other models. This makes some sense as the average appropriation level lies around 30 each period, which is easier to replicate with agents who invest exactly 30—such as in the cooperative agents model—than with agents who invest according to different strategies. The last metric, change in appropriation, has a similar fit in all ABMs. Especially for the EHSH treatment in the IND study, which comprised 50% neutral cooperative players, it makes sense that the cooperative model and the random model (which also has an average appropriation of 30 each period) have a high fit score.

**Fig 10:**
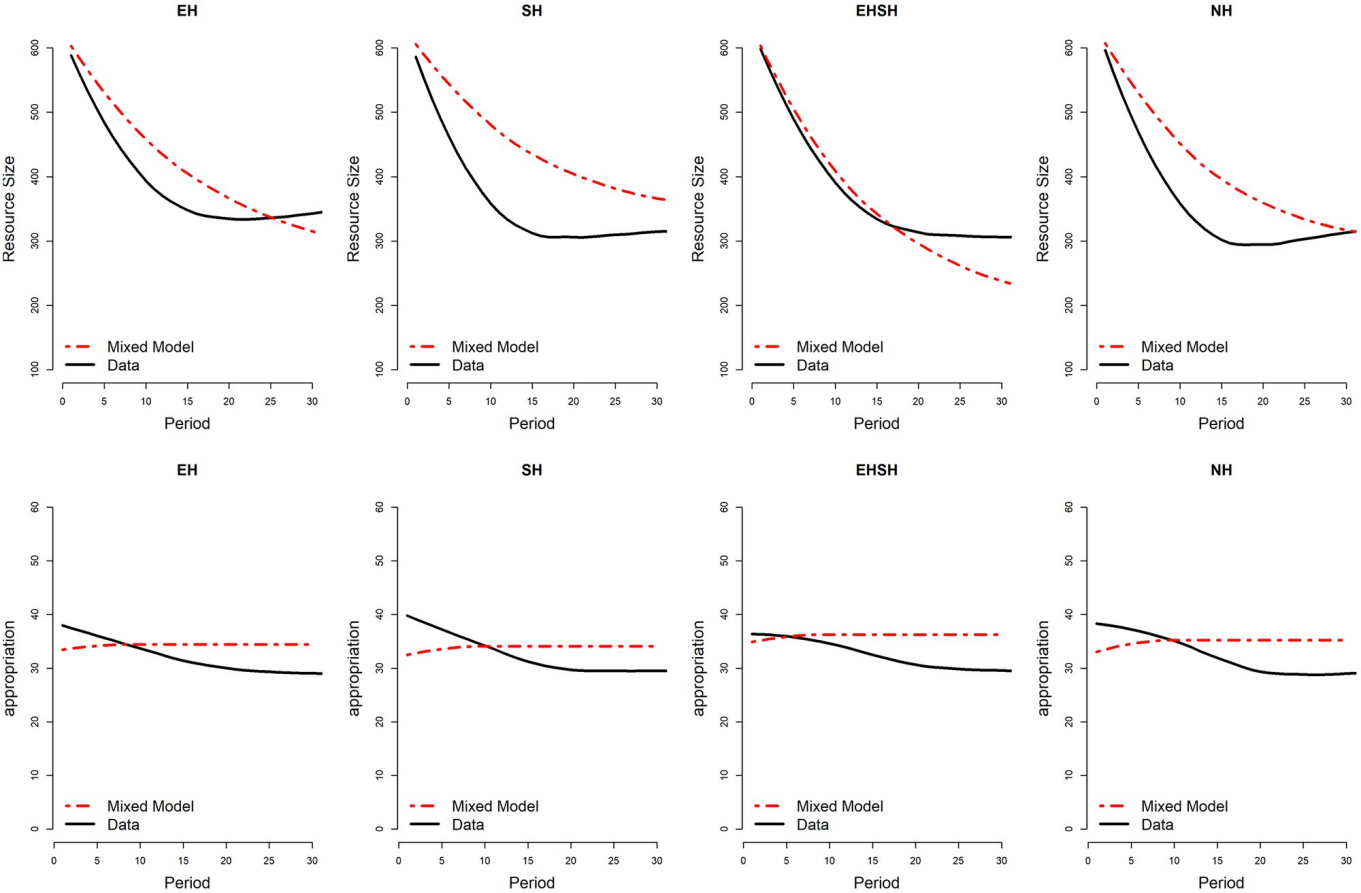
UKNL mixed model predictions for Resource Size (top row) and Appropriation Effort (bottom row).

**Fig 11:**
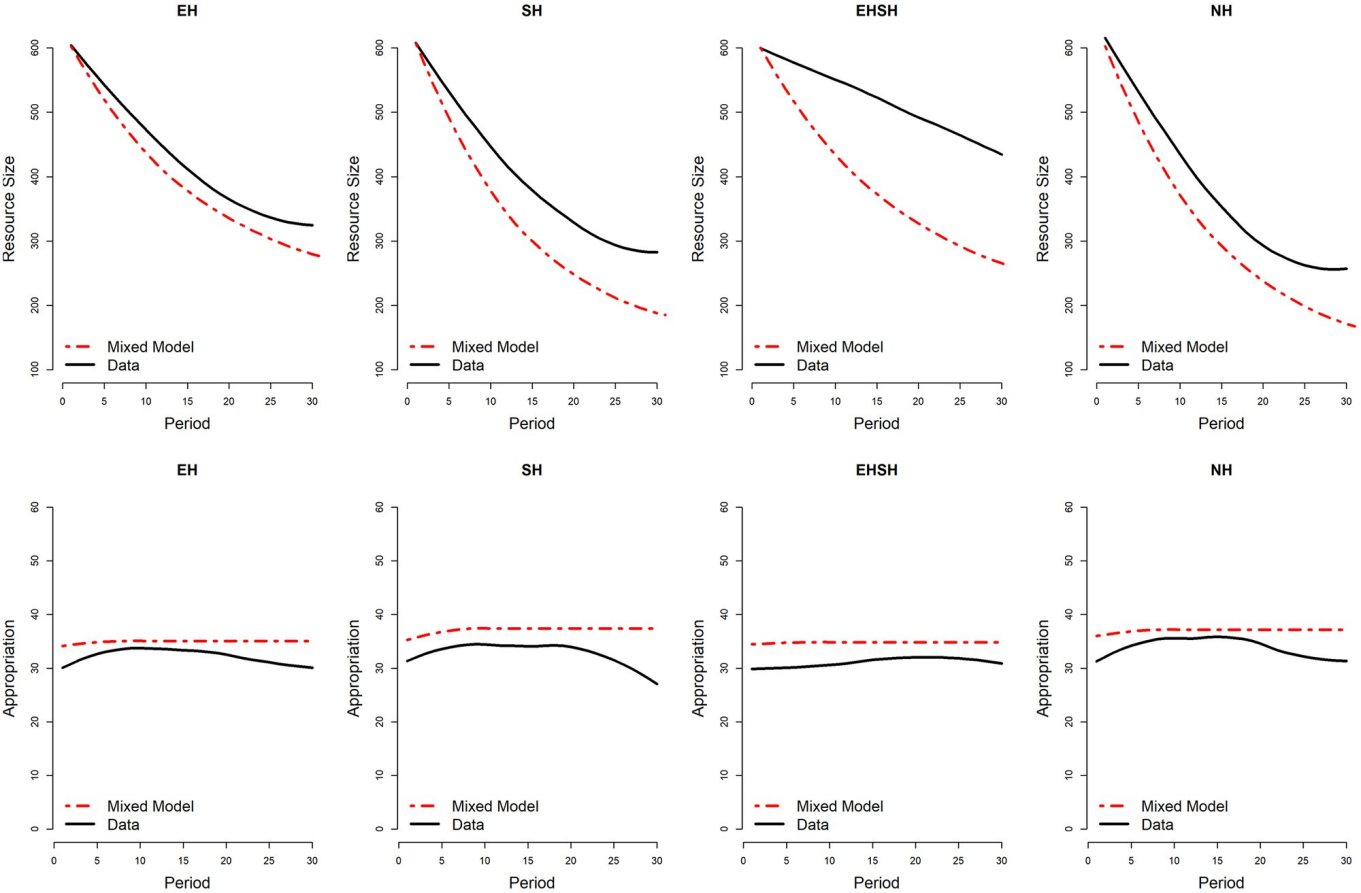
IND mixed model predictions for Resource Size (top row) and Appropriation Effort (bottom row).

To visualise how well the predictions of the ABMs fit with the actual data, Figs 10 and 11 show the mixed agent model predictions for the development of resource size and appropriation effort—being the two most important cooperation outcomes on the macro- and micro-level respectively—for the UKNL and IND study over the duration of 30 periods, compared to the actual data. The predictions are based on 1000 simulation games that were run using the *Behaviourspace* tool in NetLogo [57]. Graphs visualising the single agent model predictions versus the data can be found in S1 Fig. Graphs visualising the mixed agent model and single agent model predictions versus the data specifically for high and low endowed subjects in the EH and EHSH treatments in the UKNL and IND studies can be found in S2 Fig.

**Fig 12:**
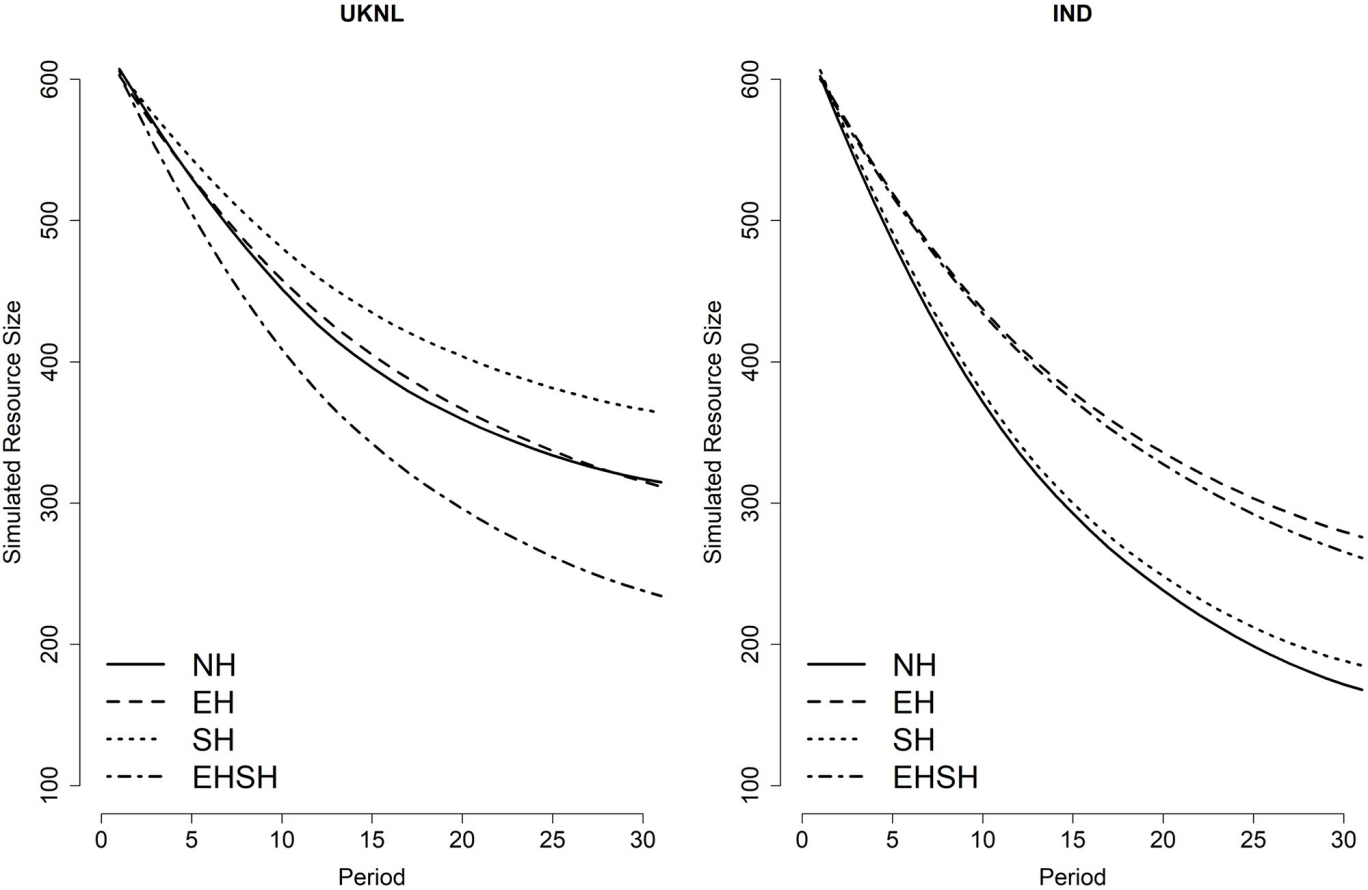
Smoothed simulated average resource size per treatment per period.

**Fig 13:**
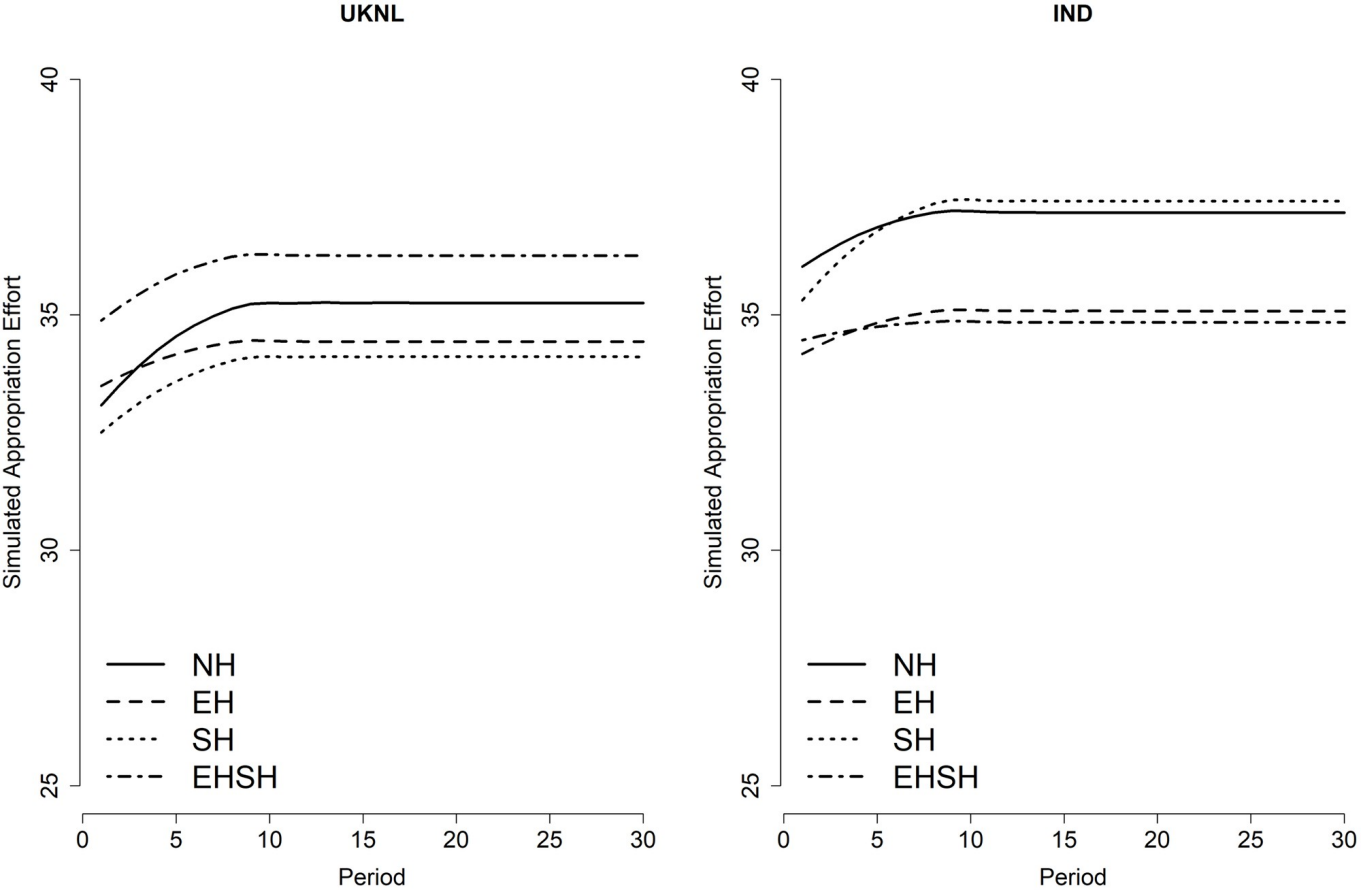
Smoothed simulated average appropriation effort per treatment per period.

The graphs confirm what the fit scores pointed out: the mixed ABM model comes close to replicating the experimental data in terms of resource size and appropriation effort over time in the Fishing Game, with the exception of resource size in the EHSH treatment of the IND study. However, the plots also show a discrepancy between the ABM models and the data, namely the position of the treatments relative to each other in terms of cooperation. This is better visualised in Figs 12 and 13, showing respectively the average simulated resource size and appropriation effort per treatment over time.

When comparing the simulated averages over time with the averages from the data, one can see that the simulations tend to make small differences between the treatments in the data even smaller, whereas bigger differences between treatments in the data tend to be enlarged in the simulations. Despite not replicating the ‘order’ of treatments in terms of cooperation perfectly, the simulations do capture some important findings.

The ABMs correctly simulated the fact that the EHSH treatment performed worst in the UKNL study, and while the EH treatment should have been simulated as best performing, the simulations do show that the EH and SH treatments do not perform worse than the NH treatment. In the real data, the differences between EH, SH and NH in the UKNL study are very small and not necessarily significant, so it is not surprising that the simulations do not replicate these differences correctly. In addition, small differences in the data can be a result of sampling variability, which means that the order of the treatments is not ‘set’ per se. Therefore, the ABM predictions are well within a reasonable understanding of the treatment effects.

In the IND study, the ABMs simulated correctly that the EHSH and EH treatments performed better than the NH treatment, and that the SH and NH treatment are very close together in terms of resource size performance. Whereas the ABMs do not make precise predictions, they do reflect the larger differences between treatments in the data which is helpful in making broad predictions on cooperation in CPRs.

## Discussion

The goal of this paper is to understand and explain differences in cooperative behaviour under heterogeneity in CPRs, by comparing and analysing experimental data using statistical methods in tandem with innovative tools such as player type classification and agent-based models. Results regarding heterogeneity and cooperation from the experiments show that sociocultural heterogeneity does not affect cooperation relative to homogeneity, and that economic heterogeneity can affect cooperation positively relative to homogeneity. These findings reject the general hypotheses in the literature that heterogeneity affects cooperation negatively, and the latter finding adds to the evidence in favour of an Olson-effect as suggested by Olson [8] and found in Van Klingeren [31]. The effect of the combination of economic and sociocultural heterogeneity is found to be positive or negative relative to homogeneity, depending on the perceived fairness of the decision-situation by the players, which is affected by the operationalisation of sociocultural heterogeneity.

The LCP classification for the complete sample shows a similar player type distribution to other experimental research on the same three player types, providing confidence in the LCP scoring approach. The LCP classification per treatment showed that the EHSH treatment in the UKNL study contained just half of the percentage of neutral cooperators and over double the percentage of conditional cooperators compared to the EHSH treatment in the IND study, while having a similar percentage of free-riders. Due to this difference, the outcome for this treatment in the IND study is better than in the UKNL study in terms of cooperation levels and thus overall CPR success. Whereas one could simply assume that worse performing treatments have more free-riders, the LCP classification showed that the difference in performance also depends on the level of conditional cooperation versus neutral cooperation. An explanation for this difference may be found in the post-experimental survey data, which revealed that subjects in the EHSH treatment of the UKNL study reported significantly lower levels of being treated fairly during the Fishing Game relative to subjects in the NH treatment—and this was not the case in the IND study. It may be the case that the combination of economic and sociocultural heterogeneity reduces the level of perceived fairness, which in turn affects the type of player someone is going to be. Important here is the operationalisation of sociocultural heterogeneity, which in the two experiments was deciding in the level of perceived fairness and thus levels of cooperation.

One way to explain the difference in the effect of sociocultural heterogeneity between the EHSH treatments by thinking of incompatibility between groups rather than differences. Given the finding that perceived fairness in the EHSH treatment in the UKNL study was significantly lower than in the IND study, it is likely that the incompatibility between two groups is more important than just the degree of sociocultural heterogeneity itself: students from two rivalling cities in India still very compatible as they share a lot of other cultural traits—especially since they go to the same university together.

The artificial nature of the identities from the MGE in the UKNL study works more polarising—as was also found in the trust games between outgroup members in Van Klingeren [32]—which leads to the percentage of conditional cooperators rather than neutral cooperators to be higher. The identification with Mumbai and Bangalore—even with some subjects being very passionate about it—did not induce the sense of rivalry between subjects as it was initially expected to. In their experiments on the effect of group identity on social preferences, Chen & Li [45] show that subjects that are matched with ingroup members act more charitably when they have a higher payoff, and show less signs of envy if they have a lower payoff.

Whereas this contributes to the understanding of the success of the EH treatments in both studies, this also holds for the EHSH treatment in the IND study: a lack of incompatibility may have created a similar or higher level of goodwill amongst participants, perhaps even *because* participants were from different groups. Future research should point out whether the incompatibility between subjects from two cities may be more significant when other sociocultural dimensions such as ethnic background, country of origin, or even continent of origin differ too. When adding the results from the IND study to the UKNL study, the conclusion of Van Klingeren [31] becomes more nuanced: not only does it matter whether economic and sociocultural heterogeneity occur at the same time, the level of polarisation and incompatibility between socioculturally heterogeneous groups also influences its impact on cooperative behaviour, by affecting people’s perceived fairness of the decision-situation and goodwill towards the other players.

Results from the analytical approach show that LCP classification is a helpful tool to translate experimental data into player type behaviour and that it helps to understand cooperation in more detail. Player type classification is found to serve as a bridge between real behaviour in experiments and simulated behaviour from ABMs. The ABMs show that experimental outcomes can to a large extent be replicated with mixed models of simple agents. Using the player type distribution of each treatment group, a mixed model including free-riders, conditional cooperators and neutral cooperators was able to replicate experimental outcomes over time relatively accurately. Agents that “played” the Fishing Game managed to capture the “signal” of the experimental outcomes, instead of noise.

This suggests that ABMs using theoretically formulated agents can be used to generate dynamic hypotheses on the development of cooperation in CPRs and to validate behavioural theories even before any experimental data has been gathered. Whereas multilevel regression models interpret data as complex behaviour, ABMs show that theoretical agents following simple rules can replicate experimental outcomes to a large extent, making ABMs a useful tool in the experimental researcher’s toolbox.

This study was limited by a number of factors. Firstly, the difference in behaviour found between the two studies could be influenced by the country it was conducted in—the IND experiment took place on another continent with a different subject pool which may not have the same exposure to experimental research as the UKNL subjects. In addition, the average age of the subjects in the IND study was 7 years lower than the average age of UKNL subjects, which may have some implications for the direct comparison between the two studies.

Moreover, there was less room in the IND study for extra games such as the other-other Dictator Game or the additional ingroup and outgroup Investment Game that were conducted in the UKNL study. Despite the expectation that these group strengthening exercises were not needed to enhance a natural identity, this could have affected subjects’ behaviour. However, when comparing each treatment between the two studies, it is clear that for the EH, SH, and NH treatments the behavioural pattern merely “shifted” on the y-axis. The shape of the resource size curves fits that of other CPR games, and the position of the EH, SH and NH treatment outcomes relative to each other in terms of macro- and micro-level cooperation did not change between the two experiments. This provides some confidence that the change in EHSH behaviour is indeed caused by the different operationalisation of sociocultural heterogeneity—rather than average age, experience with experiments, or country—which is an important result in terms of methodology and the explanation of heterogeneity effects.

Secondly, the LCP is calculated based on a linear multilevel regression, even though subjects’ behaviour over time can have a quadratic, cubic or sinusoidal pattern depending on amongst others the complexity and duration of the game—as also shown by the interaction terms of treatment effects with period and the quadratic term of period. In addition, the thresholds that divide LCP scores into player types have to be created by the experimental researcher themselves and are highly dependent on the experiment type. This makes the classification method of LCP scores less generalisable.

Thirdly, agents in the ABMs have a linear rule on what to do each round, and their actions are not influenced by the duration of the game or learning effects, whilst this may be the case in real life. However, working with ABMs will always bring with it the trade-off between simple models and fitting the data; adding more parameters to an ABM will likely increase the fit but the danger is that the models become too specific to one particular database or situation and will thus produce less generalisable results. However, even though the ABMs did not perfectly match the data, they required only minimal information in order to create patterns similar to the experimental outcomes: just by calibrating the proportions of agent types with the player types found in the data, the mixed model managed to replicate big differences in cooperative outcomes over time between the treatments. Future research can develop ABMs to include non-linear behavioural patterns and improved versions of player types, so that they can become more precise in predicting and replicating experimental outcomes.

## Conclusions

The results of this paper provide a nuanced understanding of the relation between heterogeneity and cooperation in CPRs: sociocultural heterogeneity does not impact cooperation negatively, economic heterogeneity may affect cooperation positively, and the combination of sociocultural and economic heterogeneity affects cooperation positively or negatively depending on the perceived fairness of the decision-situation. In addition, this paper concludes two important findings regarding the analytical tools used. Firstly, one can express CPR experiments in terms of three simple player types: free-riders, conditional cooperators and neutral cooperators. Secondly, agent-based models comprising theoretical, simple versions of these player types can replicate experimental outcomes using the player type distribution from the data. This means that agent-based models can be used to to generate and manipulate dynamic hypotheses about macro-outcomes of CPR games, which can lead to an increase in quality of the study of human behaviour and ultimately to a better understanding of cooperative behaviour in CPRs.

## Supporting information

S1 Text A description of the course of the experimental sessions. (PDF)

S2 Text Power analysis. (PDF)

S3 Text Inclusiveness in global research. (PDF)

S4 Text The Fishing game. A description of each stage of the Fishing Game. This description is a shorter version of game description found in Van Klingeren [31].
(PDF)

S5 Text The Investment game. This appendix comprising the explanation and details of the Investment Game used in the experiments is a copy of the one found in Van Klingeren [31] with exception of the figures showing the experimental game screens.
(PDF)

S1 Table The other-other Dictator Game. This table is a copy of the one found in the S4 Text of Van Klingeren van*_k_lingeren_p_laying*_2_020.
(PDF)

S2 Table Multilevel models. A description of the choices regarding the multilevel regressions on the macro- and micro-level of the UKNL and IND study data, and the tables showing the multilevel models.
(PDF)

S3 Table The adjusted *R*^2^. (PDF)

S4 Table Ordinal logistic regression models. Regression models on Trust and Fairness variables from the post-experimental survey.
(PDF)

S1 Fig ABM predictions. Additional graphs showing ABM predictions from the single agent ABMs compared to the data from the UKNL and IND studies.
(PDF)

S2 Fig ABM predictions for high and low endowment. Additional graphs showing mixed agent and single agent ABM predictions for high and low endowed subjects from the EH and EHSH treatments compared to the data from the UKNL and IND studies.
(PDF)
