# Supplementary material for: Using player types to understand cooperative behaviour under economic and sociocultural heterogeneity in common-pool resources: Evidence from lab experiments and agent-based models
Source: PLoS One. 2022 May 25;17(5):e0268616. doi: 10.1371/journal.pone.0268616 (PMC9132308; doi:10.1371/journal.pone.0268616)
Supplement: S1 Text — (PDF) [file pone.0268616.s001.pdf]

S1: A description of the course of the experimental sessions

Experimental Sessions

The UKNL study - conducted at the Centre for Experimental Social Sciences [CESS] at Nuffield College, University of Oxford and at the Experimental Laboratory for Sociology and Economics [ELSE] at Utrecht University between October 2018 and November 2019 comprising 344 subjects - and the IND study - conducted at the CESS Nuffield - FLAME laboratory at the FLAME University campus in Pune, India in November and December 2019 comprising 144 subjects - comprise experiments with an almost identical setup. This research, including the pre-tests, has obtained ethics clearance from the Research Ethics Committee of Department of Sociology (DREC) at the University of Oxford (Ref. SOC_R2_001_C1A_18_30), the CESS ethics committee (Ref. LE_0044) covering both the UKNL and IND study, and the sessions in the Netherlands were in addition covered by the ethical approval (Ref. FETC17-028, Buskens) of the ethical committee of the Faculty of the Social and Behavioural Sciences at Utrecht University. Written consent was obtained from all subjects in the study, including the pre-tests, before the start of each experimental session. The data were anonymised before the analyses. Both experiments are computerised laboratory experiments and were designed and programmed in z-tree [1]. The subject pools exist of (mostly) students and subjects played for real money. The average earnings for participation were 15.11 GBP/EUR and 550 INR respectively. Every session included groups for all treatments, and every subject only participated in one treatment.

General written instructions in English were handed out to the subjects at the start of the experiment. The first part of the experiment comprised a (1) one-shot Investment Game [IG] [2] to measure general trust; (2) a basic, practise version of the CPR game called 'the Fishing Game' without any treatments for three periods; and (3) a group division stage. For the UKNL study the group division was based on the MGE and for the IND study this was based on city of origin; Mumbai or Bangalore. In this stage, group bonds were strengthened through a short quiz in which group performance paid off. In addition, the UKNL study included a binary other-other Dictator Game [DG] and two one-shot IGs (once with an ingroup member and once with an outgroup member) to strengthen and test group bonds. Due to time constraints, the other-other binary DG and the additional IGs were not included in the IND study. The IND study included a comprehension check after each period of the practise Fishing Game, as it was expected that the subjects were less experienced in the type of game than the subjects in the UKNL study. In the second part of the experiment, the subjects started the Fishing Game. Prior to the start of the second part, subjects received specific instructions corresponding to their treatment. Figure 1 and Figure 2 show the sequence of the UKNL and IND experimental stages respectively. The purpose of each stage will be expanded on in the following paragraphs.

Fig 1. Sequence of UKNL experiment (Based on Van Klingeren, 2020, p.6)

		Practise		*MGE*				
IG 1	⟶	*Fishing Game*	⟶	*Quiz*	⟶	*IG 2*	⟶	*Fishing Game*
		× 3		*DG*				

Fig 2. Sequence of IND experiment

IG ⟶ *Practise Fishing Game × 3 incl. comprehension checks* ⟶ *Division Quiz* ⟶ *Fishing Game*

References

1. Fischbacher U. Z-Tree: Zurich Toolbox for Ready-Made Economic Experiments. Experimental Economics. 2007;10(2):171–178. doi:10.1007/s10683-006-9159-4.

2. Berg J, Dickhaut J, McCabe K. Trust, Reciprocity, and Social History. Games and Economic Behavior. 1995;10(1):122–142. doi:10.1006/game.1995.1027.
