# Supplementary material for: Using player types to understand cooperative behaviour under economic and sociocultural heterogeneity in common-pool resources: Evidence from lab experiments and agent-based models
Source: PLoS One. 2022 May 25;17(5):e0268616. doi: 10.1371/journal.pone.0268616 (PMC9132308; doi:10.1371/journal.pone.0268616)
Supplement: S2 Text — (PDF) [file pone.0268616.s002.pdf]

S2: Power Analysis

Using the coefficients of the interaction effect of the Economic Heterogeneity treatment with period [EH × Period] from the UKNL study - one of the effects that was expected to be replicated in the IND study as the operationalisation of the treatments was the same - a power analysis was conducted using the *simr* package [1] which was designed for power analysis of generalised linear mixed models by simulation. Using this package, an analysis was conducted to compute power against the number of levels in class - which is the numbers of groups in the current case. Figure 15 shows a plot of this analysis, and shows that with 30 groups a power of above 80% is established. This translates in $30 \times 4 = 120$ subjects that are needed.

Fig 1. Power by nr. of groups

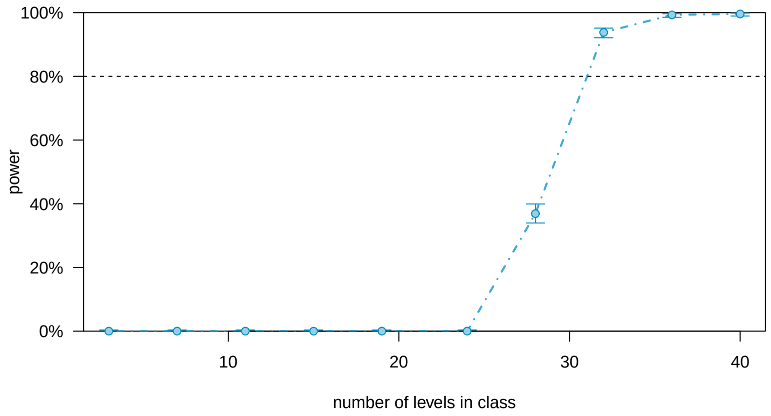

The power analysis shows that 140 subjects would be well enough to have 100% power in detecting the effect. In addition, it was calculated that a sample size of around 140 would be enough - 78 % power - to detect this effect even if its size was 0.09 instead of 0.15. The effects of Sociocultural Heterogeneity [SH] and Economic and Sociocultural Heterogeneity [EHSH] were expected to be larger and more substantive, as the IND study uses a natural identity to operationalise sociocultural heterogeneity. With these calculations in mind, 144 subjects were recruited for the IND study, that played the Fishing Game in 36 groups.

References

1. Green P, MacLeod CJ. SIMR: An R Package for Power Analysis of Generalized Linear Mixed Models by Simulation. 2016;7(4):493–498. doi:10.1111/2041-210X.12504.
