# Supplementary material for: Using player types to understand cooperative behaviour under economic and sociocultural heterogeneity in common-pool resources: Evidence from lab experiments and agent-based models
Source: PLoS One. 2022 May 25;17(5):e0268616. doi: 10.1371/journal.pone.0268616 (PMC9132308; doi:10.1371/journal.pone.0268616)
Supplement: S4 Text — A description of each stage of the Fishing Game. This description is a shorter version of game description found in Van Klingeren [31]. (PDF) [file pone.0268616.s004.pdf]

S4: The Fishing Game

A description of each stage of the Fishing Game. This description is a shorter version of game description found in Van Klingeren [1].

Appropriation of the resource

At the beginning of each period t, the appropriators all receive an endowment E of units to invest in appropriation of the resource, R. Since appropriation of the CPR is a costly activity - e.g. it takes time and requires maintenance of the boat and fishing nets - the appropriation effort $a(0 \leq a \leq E)$ represents the amount of effort an appropriator can invest in appropriation of the CPR.

The actors can choose how much they want to invest in appropriation of the resource each period. All appropriators will make their appropriation choice at the same time, without knowing what the other appropriators will do that period. They see how many fish there are in the lake and how many fish they receive per invested unit a. The appropriators all receive the same return $(\frac{4}{R_0}R_{t-1})$ per appropriation effort unit of a. The utility function for the appropriators per period is as follows:

$$U_{it} = (\frac{4}{R_0}R_{t-1})a_{it} + (E_i - a_{it})$$

U_{it} is the total utility of an appropriator i at timepoint t. In the utility function, a_{it} is the invested appropriation effort of appropriator i at timepoint t, and E_i is the endowment of appropriator i. R_0 is the original resource size of the CPR (i.e. the maximum number of fish in the lake) for which we take $R_0 = 600$. R_{t-1} is the resource size at time $t-1$. The profit per invested appropriation effort unit of a is thus dependent on the current size of the resource, relative to its original size. If $R_{t-1} = R_0$, which is the case at the first stage of the game, the return is $4 - 1 = 3$ units per invested unit of a. When $R_{t-1} < R_0$, the return will be lower than 3 units. The amount of appropriators' endowment not used for fishing is reflected by $(E_i - a_{it})$.

At the end of the period, the players see how much they invested themselves, how much profit that yielded them, and how much was invested in appropriation of the resource in total as a group. See figure 1 for a screenshot of the appropriation stage of the experiment.

Fig 1. Appropriation stage Fishing Game

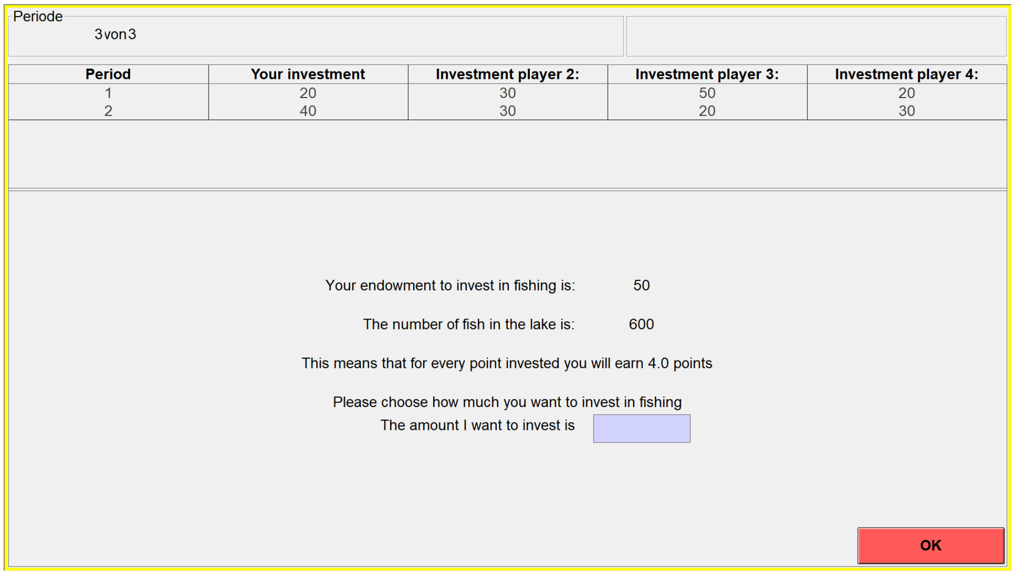

Resource renewal

Just like real natural resources, the resource in the game has a renewal rate. The renewal rate per period is modelled as follows:

$$R_t = min(600, 1.25\left(R_{t-1} - (\frac{R_{t-1}}{R_0})\sum_{i=1}^{4} a_{it}\right))$$

Here, 1.25 is the renewal rate of the resource and $R\ t$ is the resource size at timepoint t. The amount of fish in the lake is thus multiplied by 1.25 after each period. The maximum resource size is $R_t = 600$; the resource cannot grow beyond this size - this is the maximum amount of fish in the lake. The sum of appropriation efforts of all four appropriators is indicated by $\sum_{t=1}^{4} a_{it}$).

Overexploitation

The CPR is overexploited - that is, the pool of fish in the lake is smaller than in the previous period - when $R_t < R_{t-1}$, so when the resource size in timepoint t is smaller than in the previous timepoint. This happens if $\sum_{t=1}^{4} a_{it}) > 120$, because this is the limit of sustainable appropriation, based on $R_0 - \frac{R_0}{1.25}$. The CPR is thus overexploited when the four appropriators have invested on average 30 units in appropriation effort per person.[1] Investing stops being profitable if $(R_t = \frac{R_0}{4})$, so if the resource size decreased to 25 per cent of the original resource size $(R_t = 150)$, because:

$$U_{it} = (\frac{4}{600}150)a_{it} + (E_i - a_{it}) = E_i$$

When this happens, any amount the appropriator invests in appropriation of the resource will result in a return of exactly that amount, and the profit consisting of the return + the leftover endowment will thus result in $U_{it} = 50$. For example, if the appropriator invests 50 the return will be $50 + 0 = 50$, if the appropriator invests 10 the

[1] $600 - \frac{600}{1.25} = 120$

return will be $10 + 40 = 50$ etc. When the resource size drops below 25 per cent of the original size, appropriators make loss by investing in appropriation. Only when the size of the resource increases again, the multiplication of the invested unit of a will increase and fishing becomes relatively more profitable.

References

1. Van Klingeren F. Playing Nice in the Sandbox: On the Role of Heterogeneity, Trust and Cooperation in Common-Pool Resources. PloS One. 2020;15(8):e0237870. doi:10.1371/journal.pone.0237870.
