# Supplementary material for: Using player types to understand cooperative behaviour under economic and sociocultural heterogeneity in common-pool resources: Evidence from lab experiments and agent-based models
Source: PLoS One. 2022 May 25;17(5):e0268616. doi: 10.1371/journal.pone.0268616 (PMC9132308; doi:10.1371/journal.pone.0268616)
Supplement: S1 Table — This table is a copy of the one found in the S4 Text of Van Klingeren vanklingerenplaying2020. (PDF) [file pone.0268616.s006.pdf]

S6: The Other-Other Dictator Game

This table is a copy of the one found in the supporting information S4 of Van Klingeren [1].

Table 1. Other-Other Dictator Game scenario 1 (A1, B1), 2 (A2, B2) and 3 (A3, B3)

	Option given for:					
	Klees & Kandinskys		Klees		Kandinskys	
	A1		B1		B1	
Scenario 1	Klee	Kandinsky	Klee	Kandinsky	Klee	Kandinsky
	330	330	345	355	355	345
	A2		B2		B2	
Scenario 2	Klee	Kandinsky	Klee	Kandinsky	Klee	Kandinsky
	420	420	440	445	445	440
	A3		B3		B3	
Scenario 3	Klee	Kandinsky	Klee	Kandinsky	Klee	Kandinsky
	320	320	300	280	280	300

References

1. Van Klingeren F. Playing Nice in the Sandbox: On the Role of Heterogeneity, Trust and Cooperation in Common-Pool Resources. PloS One. 2020;15(8):e0237870. doi:10.1371/journal.pone.0237870.
