# Supplementary material for: Using player types to understand cooperative behaviour under economic and sociocultural heterogeneity in common-pool resources: Evidence from lab experiments and agent-based models
Source: PLoS One. 2022 May 25;17(5):e0268616. doi: 10.1371/journal.pone.0268616 (PMC9132308; doi:10.1371/journal.pone.0268616)
Supplement: S2 Table — A description of the choices regarding the multilevel regressions on the macro- and micro-level of the UKNL and IND study data, and the tables showing the multilevel models. (PDF) [file pone.0268616.s007.pdf]

S7: Multilevel Models

A description of the choices regarding the multilevel regressions on the macro- and micro-level of the UKNL and IND study data, and the tables showing the multilevel models.

Modelling and control variables

Following Van Klingeren [1], the macro-model on resource size controls for within-group levels of average general trust as measured by the IG, average age, average number of real-life acquaintances in the experimental session, average experience with game theory, percentage of students, percentage of women, and whether a session took place in the Netherlands (1) or not (0). The micro-model on appropriation effort controls for the group means as listed above, plus the individual measures of general IG trust, age, sex (female = 1), number of acquaintances, experience with game theory and being a student. In addition, the models on appropriation effort control for resource size in period $t-1$ and the sum of appropriation of others in period $t-1$. The models on resource size do not control for lagged resource size, but both the micro- and macro-level models include period by treatment interactions up to a second order polynomial to account for the dynamic nature of the treatment effects. This approach has been proven appropriate by comparing non-parametric regression splines fitted to the purged residuals using Generalised Additive Models [2, 3] with the prediction models from the multilevel regression as shown in visualisations below the multilevel regression tables. Both model types come to very similar conclusions regarding the nature of the dynamic functional forms of the treatments for both the UKNL and IND study.

Table 1. Two-level multilevel regression on Resource Size with random intercepts for groups

	(1)	(2)	(3)
Economic Heterogeneity [EH]	19.12	16.27	−10.18
	(33.73)	(32.94)	(33.18)
EH × Period	0.90***	0.88**	5.84***
	(0.24)	(0.24)	(0.86)
EH × Period^2			−0.16***
			(0.03)
EH × IND	−16.68	−20.58	−42.23
	(61.96)	(65.15)	(65.61)
EH × IND × Period	2.04***	2.68***	6.74***
	(0.44)	(0.48)	(1.68)
EH × IND × Period^2			−0.13*
			(0.05)
Sociocultural Heterogeneity [SH]	−5.73	−10.91	−20.25
	(34.07)	(33.34)	(33.58)
SH × Period	0.64**	0.63**	2.38**
	(0.24)	(0.25)	(0.86)
SH × Period^2			−0.06*
			(0.03)
SH × IND	3.13	−27.48	−26.24
	(62.15)	(64.52)	(64.97)
SH × IND × Period	0.76†	0.93*	0.69
	(0.45)	(0.47)	(1.65)
SH × IND × Period^2			0.008
			(0.05)
Economic & Sociocultural Heterogeneity [EHSH]	30.93	19.20	−8.55
	(34.45)	(33.80)	(34.04)
EHSH × Period	−0.80***	−0.80***	4.40***
	(0.25)	(0.25)	(0.87)
EHSH × Period^2			−0.17***
			(0.03)
EHSH × IND	−4.07	−2.60	−39.25
	(62.36)	(64.13)	(64.58)
EHSH × IND × Period	8.01***	7.87***	14.74***
	(0.45)	(0.47)	(1.65)
EHSH × IND × Period^2			−0.22***
			(0.05)
Mean General Trust [MGT]		35.10	35.10
		(70.40)	(70.28)
MGT × Period		3.07***	3.07***
		(0.50)	(0.42)
Period	−8.08***	−9.79***	−33.40***
	(0.18)	(0.33)	(0.67)
India [IND]	116.37*	120.50†	56.73
	(44.96)	(66.35)	(66.57)
IND × Period	−4.79***	−4.15***	7.81***
	(0.32)	(0.34)	(1.17)
IND × Period^2			−0.39***
			(0.04)
Netherlands	68.68**	87.87**	87.87**
	(26.36)	(28.41)	(28.41)
Period^2			0.762***
			(0.02)
Constant	462.88***	401.62***	527.54***
	(25.89)	(81.96)	(81.98)
Observations	14,640	14,040	14,040
Groups	122	117	117
Log Likelihood	−84,274.120	−80,854.050	−78,646.240
Akaike Inf. Crit.	168,586.200	161,758.100	157,358.500

Standard errors in parentheses.
**** $p<0.001$, ** $p<0.01$, * $p<0.05$, † $p<0.1$, two-sided*
Model 2 and 3 control for average age, game theory experience, number of acquaintances in experimental session and % female in each group

Table 2. Three-level multilevel regression on Appropriation Effort with random intercepts for groups and subjects

	(1)	(2)	(3)
Economic Heterogeneity [EH]	−0.91	−1.25	−2.16
	(1.26)	(1.38)	(1.58)
EH × Period	0.02	0.02	0.15
	(0.03)	(0.03)	(0.11)
EH × Period^2			−0.00
			(0.00)
EH × IND	−1.04	−1.92	0.85
	(2.38)	(2.80)	(3.32)
EH × IND× Period	−0.00	0.05	−0.40
	(0.06)	(0.07)	(0.32)
EH × IND × Period^2			0.01
			(0.01)
Sociocultural Heterogeneity [SH]	−0.09	0.06	0.29
	(1.28)	(1.41)	(1.60)
SH × Period	0.00	0.00	−0.03
	(0.03)	(0.03)	(0.11)
SH × Period^2			0.00
			(0.00)
SH × IND	−0.23	0.20	−0.23
	(2.39)	(2.77)	(3.27)
SH × IND × Period	−0.05	−0.04	0.00
	(0.06)	(0.07)	(0.31)
SH × IND × Period^2			−0.00
			(0.01)
Economic & Sociocultural Heterogeneity [EHSH]	−0.55	−0.56	−2.42
	(1.29)	(1.42)	(1.61)
EHSH × Period	0.02	0.03	0.29**
	(0.03)	(0.03)	(0.11)
EHSH × Period^2			−0.01*
			(0.00)
EHSH × IND	−4.74*	−4.59†	−0.23
	(2.40)	(2.75)	(3.26)
EHSH × IND × Period	0.14*	0.03	−0.63*
	(0.06)	(0.07)	(0.31)
EHSH × IND × Period^2			0.02*
			(0.01)
General Trust [GT]		−1.17	−1.23
		(1.65)	(1.62)
GT × Period		−0.11***	−0.11***
		(0.03)	(0.03)
Mean General Trust		1.15	1.49
		(3.13)	(3.07)
Period	−0.24***	−0.09***	−0.53***
	(0.02)	(0.03)	(0.09)
Period^2			0.01***
			(0.00)
India [IND]	−1.60	−2.68	−8.92**
	(1.70)	(2.72)	(2.96)
IND × Period	0.15***	0.22***	1.26***
	(0.05)	(0.05)	(0.22)
IND × Period^2			−0.03***
			(0.01)
Resourcesize (t-1)		0.02***	0.01***
		(0.00)	(0.00)
Sum appropriation others (t-1)		0.04***	0.04***
		(0.00)	(0.00)
Netherlands		−2.03†	−1.69
		(1.11)	(1.09)
Constant	36.91***	26.95***	32.20***
	(0.92)	(3.28)	(3.31)
Observations	18,080	16,895	16,895
Groups	122	117	117
Subjects	488	465	465
Log Likelihood	−71,008.380	−66,257.660	−66,248.860
Akaike Inf. Crit.	142,054.800	132,581.300	132,579.700

Standard errors in parentheses.
*** *$p < 0.001$, ** $p < 0.01$, * $p < 0.05$, † $p < 0.1$, two-sided*
Model 2 and 3 control for average and individual age, game theory experience, number of acquaintances in experimental session and % female in each group and for each individual

Model choice justification: GAM with splines

Predictions of multilevel regression models [ML] including up to a second order polynomial of 'period' versus predictions from a Generalised Additive Models [GAM] with splines.

Fig 1. ML vs GAM with splines for Resource Size UKNL (Van Klingeren, 2020, S4)

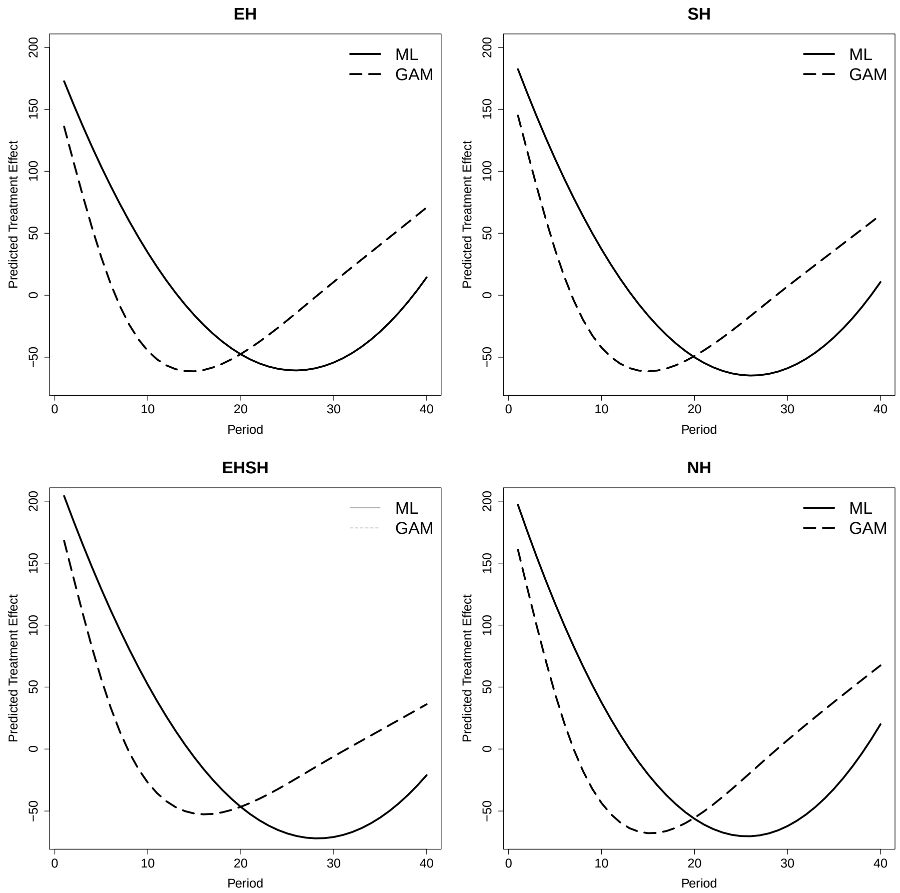

Fig 2. ML vs GAM with splines for Appropriation Effort UKNL (Van Klingeren, 2020, S5)

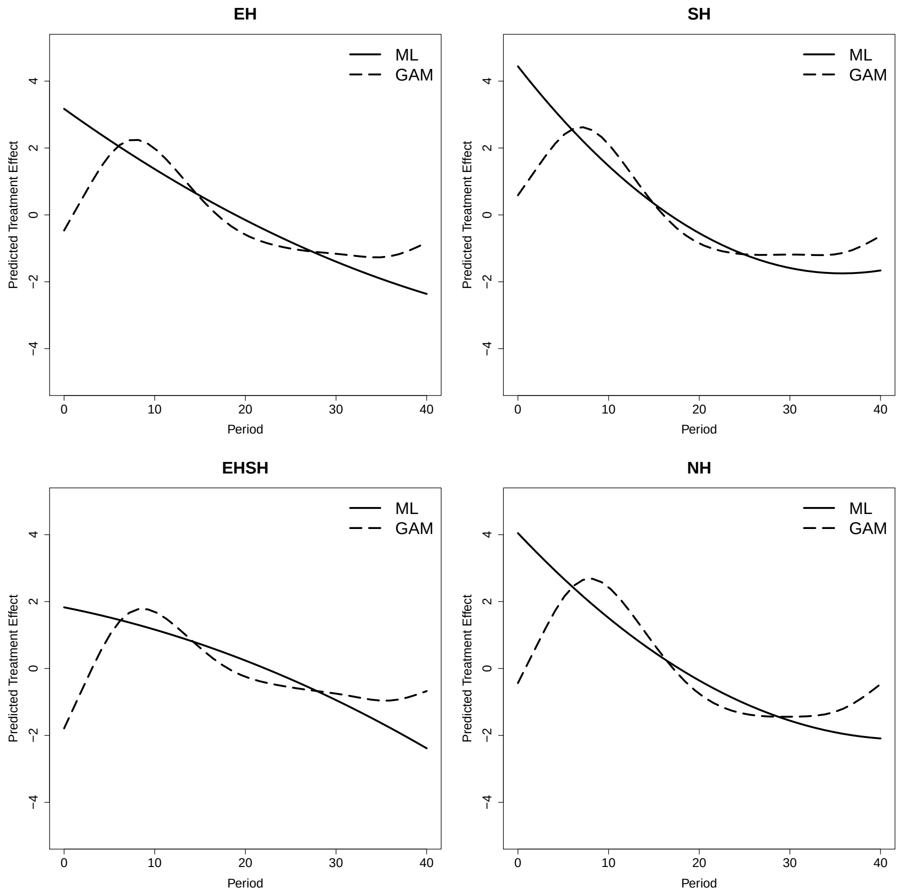

Fig 3. ML vs GAM with splines for Resource Size IND

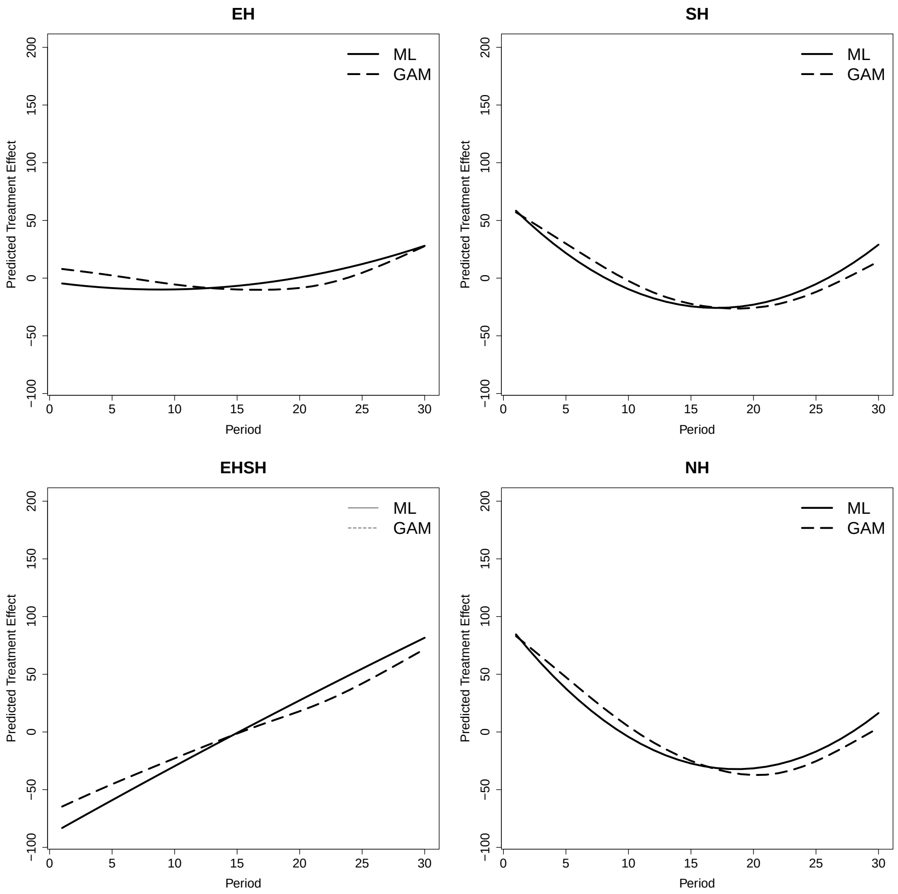

Fig 4. ML vs GAM with splines for Appropriation Effort IND

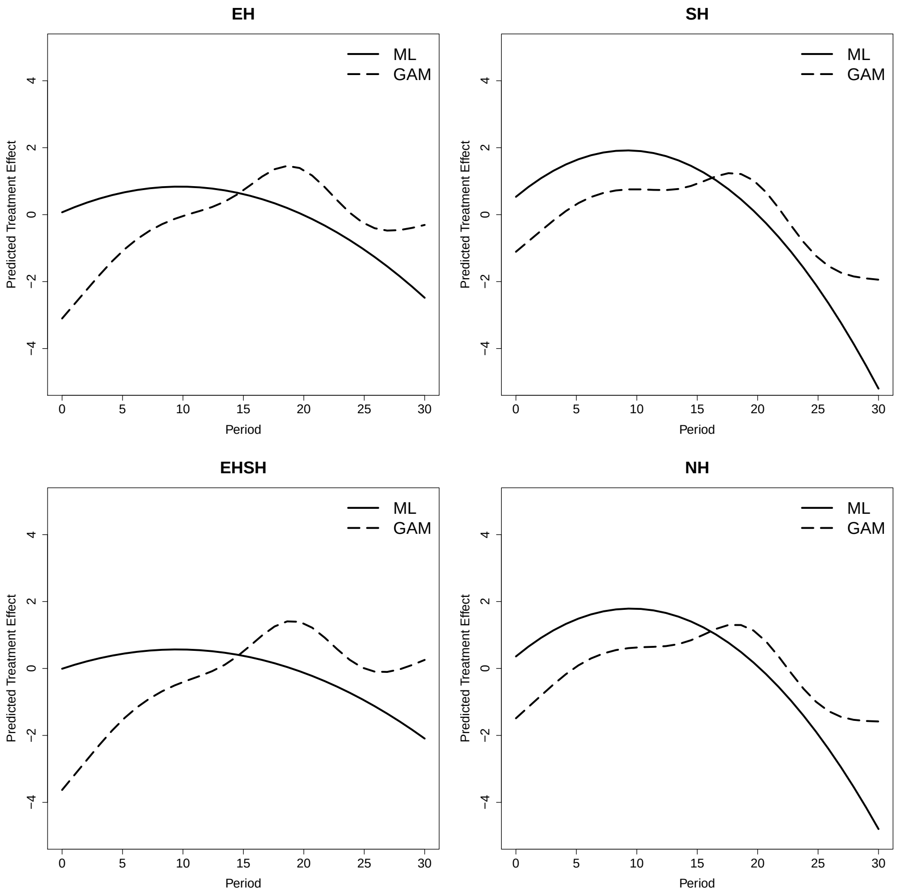

References

1. Van Klingeren F. Playing Nice in the Sandbox: On the Role of Heterogeneity, Trust and Cooperation in Common-Pool Resources. PloS One. 2020;15(8):e0237870. doi:10.1371/journal.pone.0237870.

2. Wood SN. Low-Rank Scale-Invariant Tensor Product Smooths for Generalized Additive Mixed Models. Biometrics. 2006;62(4):1025–1036. doi:10.1111/j.1541-0420.2006.00574.x.

3. Wood SN. Generalized Additive Models: An Introduction with R. Second edition ed. Chapman & Hall/CRC Texts in Statistical Science. Boca Raton: CRC Press/Taylor & Francis Group; 2017.
