# Supplementary material for: Using player types to understand cooperative behaviour under economic and sociocultural heterogeneity in common-pool resources: Evidence from lab experiments and agent-based models
Source: PLoS One. 2022 May 25;17(5):e0268616. doi: 10.1371/journal.pone.0268616 (PMC9132308; doi:10.1371/journal.pone.0268616)
Supplement: S3 Table — (PDF) [file pone.0268616.s008.pdf]

S8: The Adjusted R^2

The adjusted R^2 is calculated using

$$AdjR^2 = 1 - \left(\frac{\sum_{t=1}^{n_{mt}} (d_{mt} - s_{mt})^2)/(n_{mt} - k)}{\sum_{t=1}^{n_{mt}} (d_{mt} - \overline{d_{mt}})^2/(n_{mt} - 1)} \right)$$

where k is the number of parameters available in each ABM.

Table 1. Adjusted R^2 per ABM per treatment

		UKNL				IND			
		EH	SH	EHSH	NH	EH	SH	EHSH	NH
Mixed Model*									
	Adj. R^2 Resource Size	0.783	-0.154	0.860	0.420	0.774	0.380	-7.981	0.708
	Adj. R^2 Profit	0.527	0.398	0.829	0.670	0.605	0.427	-8.433	0.763
	Adj. R^2 Appropriation	-2.392	-0.390	-2.614	-0.601	-2.442	-3.184	-5.156	-1.152
	Adj. R^2 Change Appropriation	-0.091	-0.107	-0.086	-0.088	-0.081	-0.077	-0.075	-0.075
Cooperative									
	Adj. R^2 Resource Size	-7.652	-8.231	-6.361	-7.310	-3.669	-3.723	-2.636	-3.493
	Adj. R^2 Profit	-0.965	-3.120	-3.371	-3.006	-1.293	-2.302	-4.108	-2.002
	Adj. R^2 Appropriation	0.018	-0.513	-1.956	-0.0475	-1.616	-1.237	-3.246	-0.412
	Adj. R^2 Change Appropriation	-2.046	0.004	0.023	0.021	0.027	0.031	0.033	0.033
Free-rider									
	Adj. R^2 Resource Size	-7.652	-8.231	-6.361	-7.310	-3.667	-3.723	-2.636	-3.493
	Adj. R^2 Profit	-1.637	-2.094	-2.594	-1.785	-2.185	-3.475	-61.281	-1.742
	Adj. R^2 Appropriation	-9.384	-6.330	-7.636	-5.705	-10.581	-13.002	-46.220	-5.403
	Adj. R^2 Change Appropriation	0.018	0.004	0.023	0.021	0.027	0.031	0.033	0.033
Random									
	Adj. R^2 Resource Size	-7.003	-7.643	-5.836	-6.790	-3.280	-3.400	-2.062	-3.219
	Adj. R^2 Profit	-1.701	-1.008	-0.840	-0.997	-1.044	-0.529	-1.179	-0.542
	Adj. R^2 Appropriation	-8.568	-4.983	-6.409	-4.518	-8.277	-10.777	-15.544	-4.323
	Adj. R^2 Change Appropriation	-0.052	-0.068	-0.047	-0.049	-0.042	-0.038	-0.036	-0.036

∗ *This model consists of cooperative agents, conditionally cooperative agents and free-riding agents according to the percentages found per treatment in the LCP score calculation as apparent from figure 6 and 7.*
