# Supplementary material for: Using player types to understand cooperative behaviour under economic and sociocultural heterogeneity in common-pool resources: Evidence from lab experiments and agent-based models
Source: PLoS One. 2022 May 25;17(5):e0268616. doi: 10.1371/journal.pone.0268616 (PMC9132308; doi:10.1371/journal.pone.0268616)
Supplement: S4 Table — Regression models on Trust and Fairness variables from the post-experimental survey. (PDF) [file pone.0268616.s009.pdf]

S9: Ordinal Logistic Regression Models

Regression models on Trust and Fairness variables from the post-experimental survey.

Table 1. UKNL study: Ordinal Logistic Regression on post-experimental measures of trust (Odds Ratios). Model 1 and 2 also depicted in [1]

	(1) Trust in other players	(2) Subjective trustworthiness of others	(3) Subjective fairness of endowment division	(4) Subjective feeling of being treated fairly
EH	1.67†	1.21	0.26***	0.63
	(0.46)	(0.33)	(0.07)	(0.18)
SH	1.60†	1.13	1.67†	0.91
	(0.45)	(0.32)	(0.47)	(0.26)
EHSH	1.67†	1.06	0.18***	0.434**
	(0.47)	(0.30)	(0.05)	(0.12)
**Controls**				
Final Profit	1.19***	1.39***	1.16***	1.31**
	(0.06)	(0.07)	(0.05)	(0.06)
Age	1.04**	1.02	1.03***	1.03***
	(0.01)	(0.01)	(0.01)	(0.01)
Friends	0.97	0.82†	1.02	0.97
	(0.01)	(0.08)	(0.07)	(0.07)
Game Theory Experience	1.10	0.88	1.16	0.89
	(0.23)	(0.19)	(0.24)	(0.18)
Female	1.63*	1.11	0.91	1.04
	(0.33)	(0.23)	(0.18)	(0.21)
Netherlands	1.12	1.26	0.92	0.76
	(0.25)	(0.29)	(0.21)	(0.18)
Observations	341	341	341	341

Standard errors in parentheses.

*** $p< 0.001$, ** $p < 0.01$, * $p < 0.05$, † $p < 0.1$, *two-sided*

N = 341 due to 20 missing responses on the survey. Tables produced with Stargazer [2]

Table 2. IND study: Ordinal Logistic Regression on post-experimental measures of Trust and Fairness (Odds Ratios)

	(1) Trust in other players	(2) Subjective trustworthiness of others	(3) Subjective fairness of endowment division	(4) Subjective feeling of being treated fairly
EH	0.95	0.94	0.17***	0.41†
	(0.44)	(0.43)	(0.09)	(0.19)
SH	0.64	0.49	1.16	0.89
	(0.30)	(0.23)	(0.53)	0.42
EHSH	1.00	0.70	0.27**	0.63
	(0.46)	(0.32)	(0.13)	(0.28)
**Controls**				
Final Profit	1.00	1.01*	1.02***	1.01**
	(0.00)	(0.00)	(0.00)	(0.00)
Age	1.02	1.15†	1.01	1.11
	(0.09)	(0.10)	(0.09)	(0.09)
Friends	1.00	1.07†	1.08*	1.08*
	(0.04)	(0.04)	(0.04)	(0.04)
Game Theory Experience	0.92	1.02	1.29	1.48
	(0.31)	(0.35)	(0.45)	(0.51)
Female	1.09	1.09	0.47*	0.77
	(0.39)	(0.40)	(0.18)	(0.28)
Observations	124	124	124	124

Standard errors in parentheses.
*** $p< 0.001$, ** $p < 0.01$, * $p < 0.05$, † $p < 0.1$, *two-sided*
N = 124 due to 20 missing responses on the survey. Tables produced with *Stargazer* [2]

References

1. Van Klingeren F. Playing Nice in the Sandbox: On the Role of Heterogeneity, Trust and Cooperation in Common-Pool Resources. PloS One. 2020;15(8):e0237870. doi:10.1371/journal.pone.0237870.

2. Hlavac M. Stargazer: Well-Formatted Regression and Summary Statistics Tables. R Package Version 5.2.2.; 2018.
