# Supplementary material for: Using player types to understand cooperative behaviour under economic and sociocultural heterogeneity in common-pool resources: Evidence from lab experiments and agent-based models
Source: PLoS One. 2022 May 25;17(5):e0268616. doi: 10.1371/journal.pone.0268616 (PMC9132308; doi:10.1371/journal.pone.0268616)

S10: ABM predictions

Additional graphs showing ABM predictions from the single agent ABMs compared to the data from the UKNL and IND studies.

Fig 1. UKNL Basic Models predictions for Resource Size (top row) and Appropriation (bottom row)

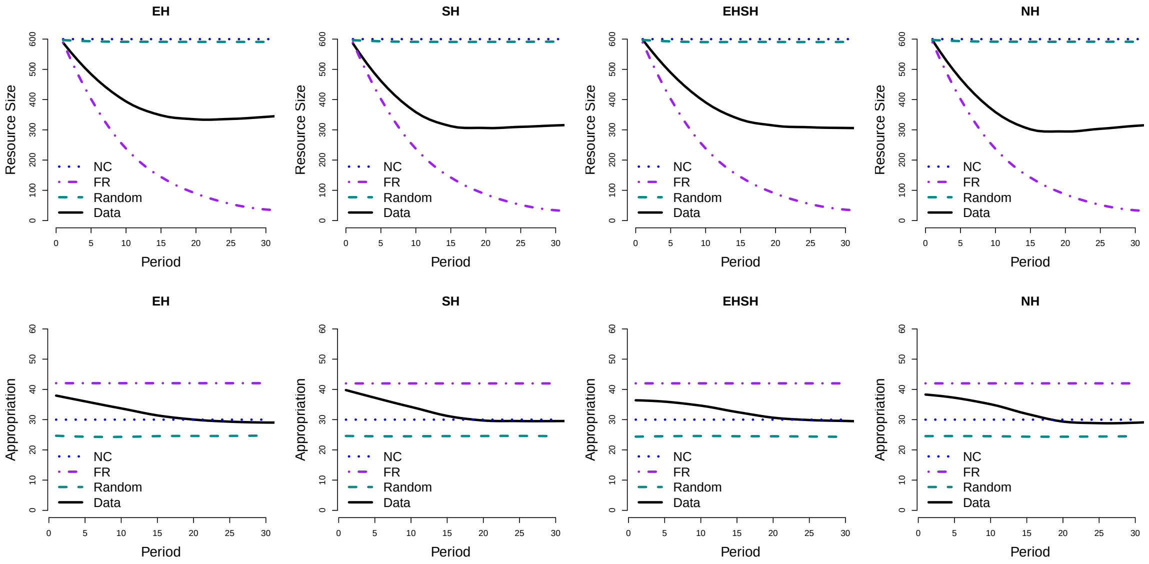

Fig 2. IND Basic Models predictions for Resource Size (top row) and Appropriation (bottom row)

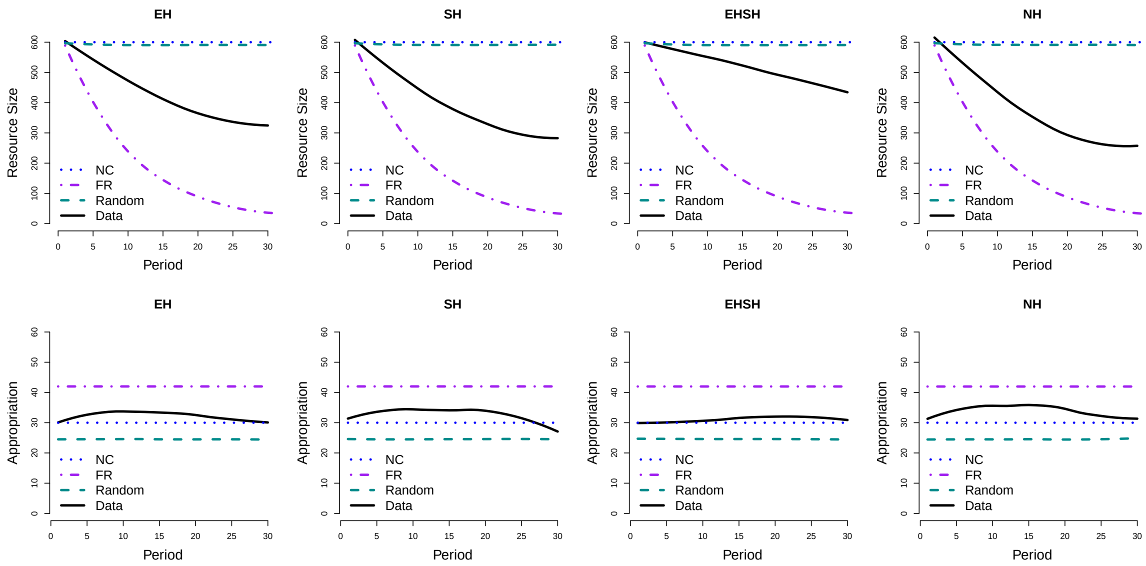

Supplement: S1 Fig — Additional graphs showing ABM predictions from the single agent ABMs compared to the data from the UKNL and IND studies. (PDF) [file pone.0268616.s010.pdf]
