# Supplementary material for: Using player types to understand cooperative behaviour under economic and sociocultural heterogeneity in common-pool resources: Evidence from lab experiments and agent-based models
Source: PLoS One. 2022 May 25;17(5):e0268616. doi: 10.1371/journal.pone.0268616 (PMC9132308; doi:10.1371/journal.pone.0268616)

S11: ABM Predictions for high and low endowment

Additional graphs showing mixed agent and single agent ABM predictions for high and low endowed subjects from the EH and EHSH treatments compared to the data from the UKNL and IND studies.

Fig 1. UKNL Mixed (top) and Basic Model (bottom) predictions for Appropriation for high and low endowments in EH and EHSH

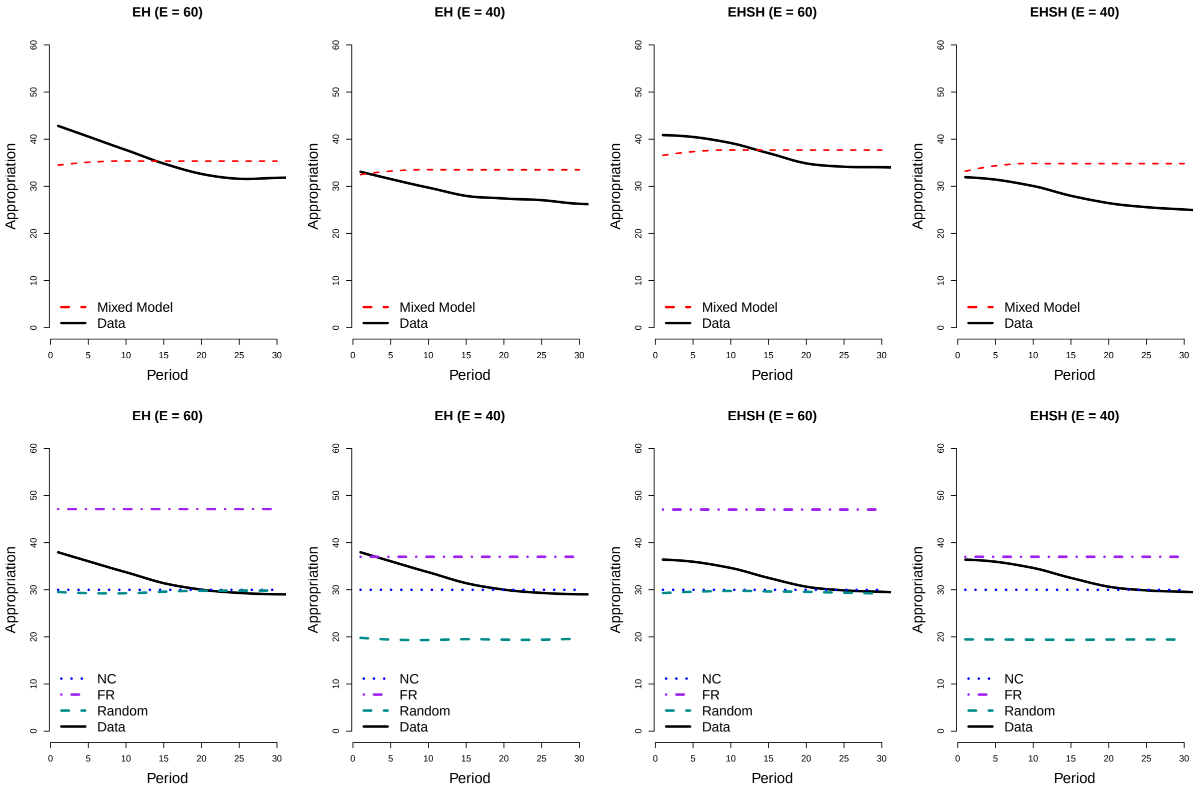

Fig 2. IND Mixed (top) and Basic Model (bottom) predictions for Appropriation for high and low endowments in EH and EHSH

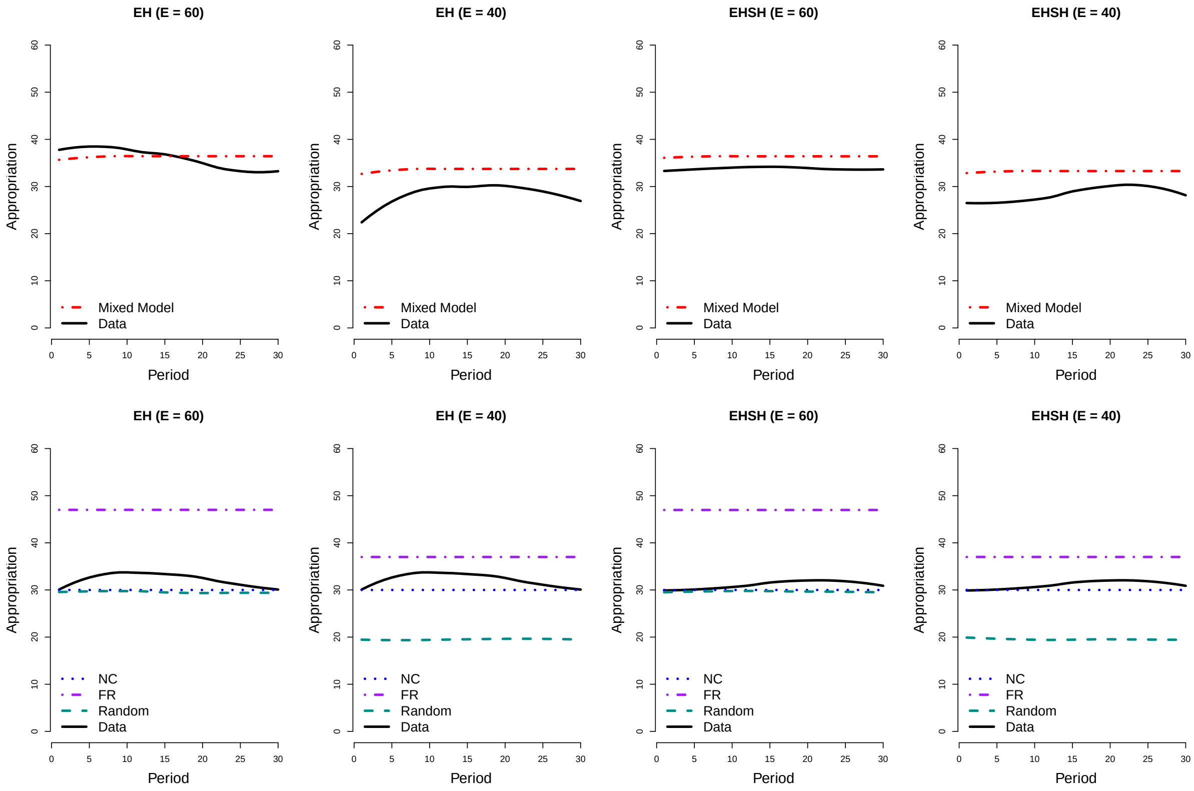

Supplement: S2 Fig — Additional graphs showing mixed agent and single agent ABM predictions for high and low endowed subjects from the EH and EHSH treatments compared to the data from the UKNL and IND studies. (PDF) [file pone.0268616.s011.pdf]
